# Classifying mental stress from eye tracking data: deep learning approaches for out-of-the-lab conditions

**DOI:** 10.1038/s41598-026-58429-7

**Published:** 2026-06-20

**Authors:** Maike Laut, Eva Dorschky, Robert Richer, Nicolas Rohleder, Bjoern M. Eskofier

**Affiliations:** 1 Machine Learning and Data Analytics Lab, Department Artificial Intelligence in Biomedical Engineering (AIBE), Friedrich-Alexander-Universität Erlangen-Nürnberg (FAU), Erlangen, Germany; 2 Chair of Health Psychology, Friedrich-Alexander-Universität Erlangen-Nürnberg (FAU), Erlangen, Germany; 3 Translational Digital Health Group, Institute of AI for Health, Helmholtz Zentrum München—German Research Center for Environmental Health, Neuherberg, Germany

**Keywords:** Computational biology and bioinformatics, Mathematics and computing, Neuroscience

## Abstract

Eye-tracking signals such as pupil diameter and gaze behavior have been widely used for stress detection, yet most approaches rely on task-specific features, controlled laboratory settings, or multimodal sensor combinations, limiting scalability in less controlled environments. This work investigates whether unimodal eye-tracking time-series data can support task-agnostic stress detection beyond static laboratory tasks. We analyze stress classification across two complementary datasets: a virtual reality goalkeeper task with moderate visuomotor activity and stable recording conditions, and a virtual job interview dataset reflecting less controlled settings with uncalibrated signals. The results show that these signals alone contain informative patterns related to stress-associated autonomic and oculomotor responses. Under favorable conditions, performance reaches up to ${95.98}\%$ macro-averaged F1-score. At the same time, performance varies substantially across datasets, indicating that effective learning depends strongly on data quality, calibration, signal characteristics, and task design. Overall, the findings demonstrate the potential of unimodal eye tracking as a lower-burden alternative to more complex multimodal systems, while highlighting that reliable stress detection is fundamentally conditioned by the interplay of data, signal representation, and modeling approach.

## Introduction

Stress arises throughout life, from the academic and social demands of childhood to the responsibilities of adulthood^1^. Physiologically, it is mediated by two pathways: the sympathetic-adrenal-medullary (SAM) system and the hypothalamic-pituitary-adrenal (HPA) axis. The SAM system triggers rapid fight-or-flight responses via adrenaline and noradrenaline, whereas the HPA axis regulates longer-term adaptation through cortisol^2^. Although essential for survival, chronic activation can increase the risk of cardiovascular disease, depression, and metabolic disorders^1,3,4^. Reliable stress detection is therefore important for timely intervention and prevention^5^. Traditional methods such as psychometric questionnaires or biomarker analysis are informative but impractical for continuous use^6^. Wearable sensing and machine learning (ML) offer a non-invasive alternative based on physiological signals including electrodermal activity (EDA), heart rate, speech, posture, and pupillometry^6–11^.

Compared with wrist-worn modalities, eye tracking (ET) provides complementary insights into cognitive and perceptual processes. Pupil diameter (PD) is the most widely studied marker: sympathetic activation during arousal or cognitive effort induces dilation, whereas parasympathetic activity promotes constriction^12^. Other oculomotor signals show similar sensitivity to stress and workload: blink rate often decreases under cognitive load but increases with emotional stress, and demanding tasks produce shorter, more frequent fixations^13^. Although influenced by luminance and fatigue^14^, these markers remain sensitive to mental states. The growing integration of ET into virtual reality (VR) headsets and mobile devices therefore makes it attractive for stress detection, particularly in human–computer interaction and home-based VR scenarios, where gaze behavior and pupil responses can be used to adapt task demands, for example by adjusting task difficulty or providing real-time feedback when increased cognitive load or stress is detected^15^.

**Fig. 1:**
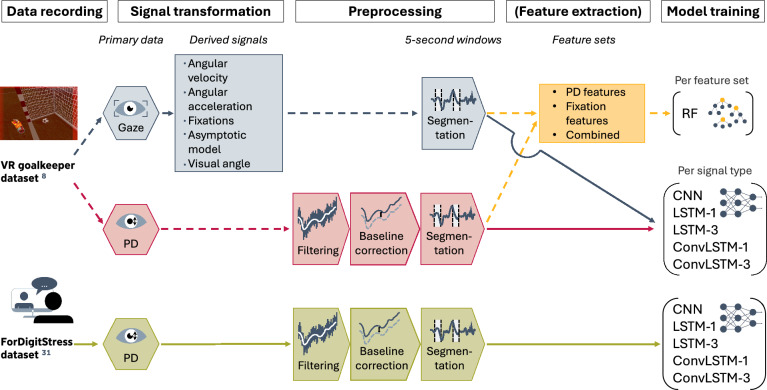
Overview of the proposed stress classification pipeline, illustrating dataset-specific preprocessing and model pathways, as well as the flow of information indicated by color-coded arrows. Raw eye-tracking (ET) recordings provide time-series signals of pupil diameter (PD) for both datasets and, for the virtual reality (VR) goalkeeper dataset, calibrated gaze coordinates. From these gaze signals, additional time-series representations are derived, including visual angle, angular velocity, angular acceleration, fixations, and asymptotic-model outputs. PD signals undergo dataset-specific preprocessing, including artifact removal, filtering, and divisive baseline correction, followed by segmentation into fixed-length five-second windows. While the overall preprocessing steps are conceptually consistent, their implementation differs between datasets due to variations in recording conditions (e.g., binocular vs. monocular tracking, eye tracker calibration availability, and sampling rate). The resulting windows form labeled stress and non-stress samples. Three modeling pathways are evaluated: (i) a feature-based approach applied to the VR goalkeeper dataset, where task-agnostic features (15 PD statistics and 3 fixation features) are extracted and classified using a random forest (RF), indicated by yellow dashed arrows; (ii) a time-series-based approach on the VR goalkeeper dataset, where individual normalized ET signals—either PD (blue arrows) or individual gaze-derived signals (red arrows)—are used separately as input to deep learning models (CNN, LSTM, and ConvLSTM); and (iii) a time-series-based approach on the ForDigitStress dataset, where only PD is available and used as input to the same model architectures (green arrows). For the recurrent architectures, LSTM-1 and ConvLSTM-1 denote models with a single LSTM or ConvLSTM layer, respectively, while LSTM-3 and ConvLSTM-3 denote models with three stacked layers of the corresponding type. Model training and evaluation follow a nested, leave-one-subject-out cross-validation scheme with inner-loop hyperparameter optimization, enabling assessment of generalization to unseen participants.

In current research, stress detection systems predominantly follow a multimodal approach, combining ET with physiological signals such as EDA, electrocardiography (ECG), or facial temperature to improve classification performance^16–20^. These approaches typically achieve high accuracies, often above ${80}\%$ to ${90}\%$, by leveraging complementary information across modalities. However, this improved performance comes at the cost of increased system complexity, additional hardware requirements, and more demanding data acquisition, which restrict their suitability for scalable and everyday deployment. In contrast, unimodal ET-based approaches rely solely on gaze and pupillary signals and represent a complementary, low-burden option that prioritizes scalability and ease of deployment, for example in resource-constrained or interaction-monitoring settings, rather than maximum predictive performance. Existing studies using PD, fixation, or blink features report accuracies of ${70}\%$ to ${87}\%$ in tasks such as Stroop tests, driving simulations, and VR-based assessments^7,21,22^. However, unimodal approaches remain comparatively scarce, and existing methods often depend on task-specific feature design or high-quality eye-tracker calibration, limiting their generalizability, particularly under less controlled conditions.    

Early ET-based stress recognition relied on hand-crafted features such as saccade frequency, fixation duration, or PD variability^16^. Recently, deep learning (DL) methods have gained attention because they learn representations directly from raw data and reduce reliance on task-specific feature engineering. Convolutional neural network (CNN), long short-term memory (LSTM), and hybrid convolutional long short-term memory (ConvLSTM) architectures show strong results in driver monitoring, VR-based stress tasks, and multimodal systems^23–25^. Some studies report accuracies above ${90}\%$ using PD alone^24^. However, other work shows that ET-only DL models can still underperform compared to multimodal approaches^26^. Overall, neural networks for unimodal ET-based stress detection remain underexplored, with most work limited to controlled laboratory settings with artificial stressors, restricted movement, and low label uncertainty^18,27–29^.

As a result, the behavior of these models under less controlled conditions remains insufficiently understood, particularly in the presence of heterogeneous recording setups, variations in calibration and signal quality, and increased variability in gaze behavior. Table 1 provides an overview of representative ET-based stress detection studies using DL. The table summarizes stress paradigms, input signals, dataset sizes, and reported performance, highlighting the research gaps addressed in this work.

Because task design strongly shapes gaze behavior, evaluating ET-based stress detection requires diverse contexts. We therefore analyze two complementary datasets representing different challenges under less controlled and semi-naturalistic conditions: (1) a VR goalkeeper dataset introducing movement and visuomotor demands and released with this work, and (2) the ForDigitStress virtual job interview dataset, which induces psychosocial stress under less controlled conditions and includes uncalibrated ET data^26,30^. This setup enables a cross-context comparison across distinct recording conditions and stress paradigms, rather than formal external validation on a harmonized test cohort.

**Table 1:** Representative eye-tracking (ET)-based stress detection studies and key research gaps addressed in this work. For each study, only the best-performing model and performance are reported. ET-only results are shown in brackets and marked with ($^{*}$), indicating that the reported performance was obtained using an ET-only modality subset and the corresponding participant subset.

Study	Stressor	ET / additional signals	#P	Best model	Performance	Key limitation
Mou et al.^23^	Sim. Driving	PD$^{*}$, Gaze$^{*}$, Blinks$^{*}$, Veh., Env.	22	Attention CNN–LSTM	Acc. 95.5% (92.2%)$^{*}$	Stress labels not physiologically validated
Vaitheeshwari et al.^25^	VR Sim. Driving	PD$^{*}$, Blinks$^{*}$, HRV, EDA	20	LSTM	Acc. 99.7% (89.3%)$^{*}$	Sim. scenario, Unvalidated stress, Participant-level leakage risk
Jyotsna et al.^21^	Videos	PD, Gaze, Blinks	6	LSTM+RF	Acc. 86.4%	Personalized
Rescio et al.^31^	TSST, Math, MIST, SCWT	Blinks, EDA, HR	20	CNN	Acc. 96.9%	Controlled lab setting, Limited ET signal diversity
Zhao et al.^24^	Sim. Driving	PD, Gaze	44	CNN–LSTM	Acc. 95.39%	Sample-level split (no subject separation)
Heimerl et al.^26^	Virtual Interview	AE pupil$^{*}$, HR, Pose, Audio, AU, EDA	40 (19)$^{*}$	LSTM	Acc. 91.7% (70.2%)$^{*}$	AE-derived features, Multimodal dependency

This work						Contributions
VR Goalkeeper dataset	VR sports task	PD, Gaze dynamics	27	ConvLSTM	F1 95.98%	*Task-agnostic,* *Moderate movement*
ForDigitStress dataset	Virtual interview	PD (monocular)	15	CNN	F1 57.77%	*Unimodal,* *Naturalistic stressor*

Sim.: simulated, VR: virtual reality, TSST: Trier Social Stress Test, MIST: Montreal Imaging Stress Task, SCWT: Stroop Color–Word Test, PD: pupil diameter, AE: autoencoder, HRV: heart rate variability, HR: heart rate, EDA: electrodermal activity, AU: facial action units, Veh.: vehicle data, Env.: environmental data, Acc.: accuracy, F1: F1-score, #P: number of participants

To advance ET-based stress detection toward less controlled settings, we investigate a DL pipeline operating on raw ET time series, including established signals such as PD and fixations^23–25^ as well as less explored signals such as gaze velocity, acceleration, and position. We evaluate binary stress classification using three architectures — CNN, LSTM, and ConvLSTM — and relate their performance to a task-independent feature-based baseline^7^. Rather than identifying a single best-performing model, this comparison is used to analyze how different signal characteristics interact with model architectures and to what extent stress-related information can be captured directly from raw time-series data. The selected architectures are widely used for physiological time-series analysis because they capture temporal patterns while maintaining moderate model complexity, which suits the relatively small datasets typical in ET-based stress studies. Moreover, CNN-based, LSTM-based and hybrid convolutional-recurrent architectures currently represent the dominant deep learning paradigms in ET-based stress detection research (Table 1). The present study therefore focuses on a controlled comparison of established architectures and underexplored ET signal representations rather than on maximizing performance through increasingly specialized model designs.

Together, these contributions provide a systematic analysis of how stress-related information in ET signals can be captured under diverse recording conditions and how this depends on data quality, calibration, signal characteristics, task design, and their interaction with model architectures, highlighting both the potential and the practical constraints of unimodal ET-based stress detection. Figure 1 summarizes the approach.

## Methods

This section describes the datasets, preprocessing steps, feature extraction procedures, model training, and evaluation strategy used for stress classification from ET data. Figure 1 provides an overview of the complete processing pipeline. We first introduce the VR goalkeeper dataset, which is released with this work, and describe its preprocessing, extracted ET signals, feature-based baseline, and DL training procedure. We then present the ForDigitStress dataset^26^ and the corresponding preprocessing, model training, and evaluation setup. Together, the datasets provide complementary settings for assessing ET-based stress detection under different recording conditions and task contexts.

### VR goalkeeper dataset

The VR goalkeeper dataset released with this publication contains recordings of PD and gaze behavior during a virtual penalty kick scenario. Thirty football players (24 male, 6 female; age: M = 22.3, SD = 2.7 years) completed 20 penalty kicks in a non-stress condition and 20 penalty kicks in a stress condition, which was induced using a working memory task, performance feedback, and competition. ET data were recorded using an HTC Vive Pro Eye headset (HTC Corporation, Taoyuan, Taiwan) with the Tobii Pro VR ET system (Tobii, Danderyd, Sweden), operating at 120Hz with five-point calibration and an effective sampling rate of 90Hz. Because all participants experienced the same virtual environment and display conditions, illumination remained constant across recordings. ET recordings were successfully obtained for 27 participants; three recordings were excluded due to hardware-related acquisition failures. The resulting dataset therefore contains binocular recordings from 27 players comprising 1,080 penalty kicks. The stress induction procedure and its physiological validation using salivary $\alpha$-amylase are described by Stoeve et al.^7^, who reported significantly elevated stress responses during the stress condition. All participants provided written informed consent prior to the recordings. The study was approved by the ethics committee of Friedrich-Alexander-Universität Erlangen-Nürnberg (Erlangen, Germany; Re-No. 106_13B), and all methods followed the Declaration of Helsinki.

Preprocessing followed the pipeline of Stoeve et al.^7^. Blinks and invalid samples were removed using TobiiXR flags with a 50ms margin^32^. Additional dilation-speed, trend-line deviation, and sparsity filters were applied to reduce measurement noise and eyelid occlusion artifacts^33^. Binocular PD was then averaged, using inter-eye correlation to replace missing samples^33^, and the remaining short gaps were interpolated. Finally, one five-second window from the preparation phase before each kick was extracted, resampled to 450 samples and min-max normalized, yielding 540 stress and 540 non-stress windows across 27 participants; none were excluded during preprocessing.

### Feature-based stress classification

**Table 2:** Task-agnostic features used in the feature-based stress classification approach, grouped into pupil diameter (PD) statistics and fixation characteristics. Details on feature computation are provided in^7^.

Feature category	Metrics
PD statistics (15)	Mean, Median, Standard deviation, Variance, Skewness, Kurtosis, Maximum value, Minimum value, Range, 1st quantile, 3rd quantile, Harmonic mean, Samples until maximum, Slope (first half), Slope (second half)
Fixation characteristics (3)	Average fixation duration, Fixation durations, # fixations

In contrast to our previous task-specific approach^7^, we extracted a task-independent feature set of PD statistics and fixation measures (Table 2). Fixations were detected using the velocity-based asymptotic model of Duchowski *et al.*^32^. A random forest (RF) classifier was trained using (i) PD statistics, (ii) fixation features, or (iii) both. Model evaluation followed a nested leave one-subject-out cross-validation (LOSO-CV) scheme. In the outer loop, LOSO-CV was used to assess generalization to unseen participants, while hyperparameter optimization was performed in the inner loop using stratified five-fold cross-validation (CV) and Bayesian optimization (Optuna), maximizing the macro-averaged F1-score,

1 $$\begin{aligned} F_{1,\text {macro}}= \frac{1}{C} \sum _{i=1}^{C} \frac{2 \cdot \text {precision}_i \cdot \text {recall}_i}{\text {precision}_i+\text {recall}_i}, \end{aligned}$$

where $C$ denotes the number of classes. Univariate feature selection was applied within the inner cross-validation loop to avoid information leakage and to reduce dimensionality^34^. Hyperparameter ranges are listed in Supplementary Table S1. Performance was evaluated using accuracy and F1-score, and the number of selected features served as an indicator of redundancy. The same nested evaluation and optimization framework was later applied to all DL models to ensure a comparable experimental setup. Feature-selection frequencies across outer LOSO-CV folds were additionally analyzed for the combined feature set and are reported in Supplementary Fig. S2.

### Time-series-based stress classification

DL enables learning patterns directly from raw ET signals^23–26,35^. We evaluated CNN, LSTM, and ConvLSTM models on raw PD and gaze-derived time series (visual angle, velocity, acceleration, position, fixations, and asymptotic-model output), padding shorter signals to achieve uniform length. The individual signals and their preprocessing are described below.

**PD** was normalized using divisive baseline correction:

2 $$\begin{aligned} \Delta \textrm{PD} = \frac{\textrm{PD} - \textrm{median}_{b}}{\textrm{median}_{b}} \, , \end{aligned}$$

where $\textrm{median}_{b}$ denotes the baseline median PD computed over a one-second artifact-free window^14,36^.

The first 30s were discarded to avoid instruction-related fluctuations^7^. This normalization reduces inter-individual offsets and slow pupil-size drifts caused by physiological variability or stable illumination differences.

**Visual angle** describes the angular change in gaze direction relative to head position. Let $\textbf{p}_i=(x_i,y_i,z_i)$ denote the gaze intersection points (GIP) at sample $i$ and $\bar{\textbf{h}}$ the mean head position within the window. The eye-direction vector is $\textbf{v}_i=\textbf{p}_i-\bar{\textbf{h}}$. The instantaneous visual angle $\theta _i$ between consecutive samples is

3 $$\begin{aligned} \theta _i = \cos ^{-1}\!\left( \frac{\textbf{v}_i \cdot \textbf{v}_{i+1}}{\Vert \textbf{v}_i \Vert \Vert \textbf{v}_{i+1} \Vert } \right) , \quad i = 0,\dots ,n-2 , \end{aligned}$$

where $n$ denotes the number of samples in the window, $\cdot$ the dot product, and $\Vert \cdot \Vert$ the Euclidean norm. Using the mean head position provides a stable reference because eye movements are typically faster than head movements^37,38^. Angles were computed in radians and converted to degrees.

**Angular velocity** quantifies the rate of change of the visual angle and was computed using a finite impulse response (FIR) smoothing filter^38^:

4 $$\begin{aligned} \dot{\theta }_i = \frac{1}{\Delta t} \sum _{j=0}^{k} \theta _{i+j} h_j, \quad i = 0,\dots ,n-k-1 , \end{aligned}$$

where $\Delta t$ denotes the sampling interval, $h_j$ the FIR coefficients, $k$ the filter length, and $n$ the number of samples in the window.

**Angular acceleration** captures rapid changes in angular velocity and was computed using a differential high-pass FIR filter^38,39^:

5 $$\begin{aligned} \ddot{\theta }_i = \frac{1}{\Delta t} \sum _{j=0}^{k} \dot{\theta }_{i+j} g_j, \quad i = 0,\dots ,n-k-1 , \end{aligned}$$

where $g_j$ denote the FIR coefficients.

**Fig. 2:**
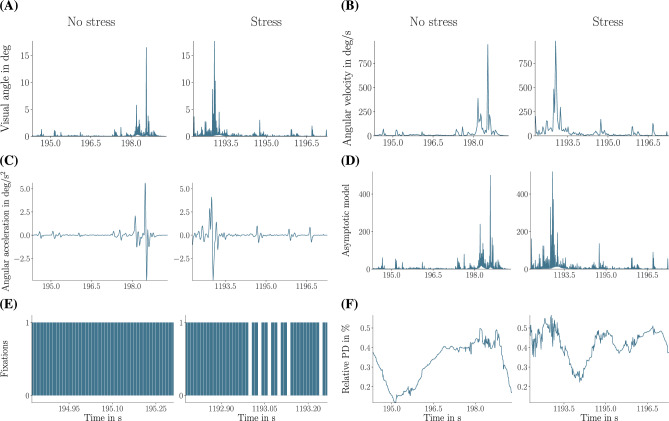
Representative time-series signals from one participant in the virtual reality (VR) goalkeeper dataset. Each panel (**A**–**F**) shows stress and non-stress windows for a different eye-tracking (ET) signal used as model input. Panels (**A**–**D**,**F**) show the full 5 s window, while panel (**E**) shows a 500 ms snippet due to the shorter time scale of fixation events.

**Fixations** were detected when angular velocity fell below ${130}^{\circ }s$^32^. The sequences were encoded as numerical vectors for neural network input. Figure 2 illustrates example time-series signals from a single participant in the VR goalkeeper dataset, where each signal represents one five-second window of a specific modality.

### Model training for the virtual reality (VR) goalkeeper dataset

Models were trained and evaluated using nested subject-wise LOSO-CV. In the outer loop, windows from one participant formed the test set and the remaining participants formed the training data. Within the inner loop, hyperparameters were optimized using Bayesian optimization with Optuna (50 trials)^40^. Each trial was evaluated using stratified five-fold CV, and model selection was based on the mean macro-averaged F1-score across the inner folds (Equation 1). Hyperparameter search spaces are listed in Supplementary Tables S2–S7, including learning rate, focal-loss parameters ($\alpha$, $\gamma$)^41^, network architecture parameters (e.g. hidden units, kernel size, pooling size, and number of segments), and regularization parameters such as dropout and L1/L2 penalties. The same nested evaluation and optimization strategy was used for the feature-based baseline (see Section Feature-based stress classification), ensuring a comparable experimental setup across methods.

The best hyperparameters were used to retrain each model on the full outer training set and evaluate it on the held-out test participant. Because each test fold contains data from a previously unseen participant, this protocol evaluates generalization across individuals rather than subject-specific patterns. Models were trained with Adam^42^ for 100 epochs (batch size 32), using early stopping (patience 20) and learning-rate reduction (patience 15, factor 0.5). Model selection was based on the epoch with the highest validation macro-averaged F1-score, and the corresponding weights were restored for final evaluation. In practice, models converged well before 100 epochs (see loss curves in Supplementary Fig. S1). Training was performed with a single NVIDIA RTX 3080 GPU (10 GB VRAM) and Intel Xeon CPUs. Computational complexity was evaluated separately via forward-pass measurements on a local machine (Apple M3 Pro chip, 36 GB RAM; see Supplementary Section D.4).

We compared CNN, LSTM, and ConvLSTM architectures commonly used in stress detection^23–25^. These models are well suited for physiological time-series analysis: CNNs capture local temporal patterns through convolutional filters, LSTMs model sequential dependencies, and ConvLSTMs combine both mechanisms. Their moderate complexity further makes them appropriate for the relatively small datasets typical of ET-based stress studies. The CNN architecture comprised three 1D convolutional layers with rectified linear units (ReLU) activation, max pooling, and dropout, followed by a dense softmax output layer. For the recurrent architectures, we evaluated both single-layer and three-layer variants. Specifically, LSTM models with one and three stacked layers are denoted as LSTM-1 and LSTM-3, respectively, and ConvLSTM models as ConvLSTM-1 and ConvLSTM-3. The LSTM models incorporated batch normalization and a regularized dense softmax output layer^43,44^. The ConvLSTM architectures combine convolutional and recurrent processing within each layer. Input sequences were segmented (with the number of segments optimized via Optuna), and dropout was applied before the final softmax layer^45,46^.

To complement the descriptive evaluation, inferential statistical comparisons between the best-performing DL models and corresponding feature-based baselines were performed on outer-fold macro-averaged F1-scores. Specifically, we compared the best overall DL model with the combined-feature RF baseline and the best PD-based DL model with the PD-feature RF baseline. Because the paired fold-wise differences may not follow a normal distribution and the number of held-out participants was limited, paired comparisons were conducted using the non-parametric Wilcoxon signed-rank test. Multiple statistical comparisons increase the risk of inflated Type I error rates; therefore, p-values were adjusted using the Holm correction method, which controls the family-wise error rate while providing greater statistical power than the standard Bonferroni correction. Additional details regarding the statistical analysis and effect size estimation are provided in the Supplementary Section D.2.

### ForDigitStress dataset

To cover a broader range of stress scenarios, we included the ForDigitStress dataset^26^, which captures psychosocial stress during a simulated virtual job interview. Stress labels were derived from self-reports, interview content, and salivary cortisol, providing both psychological and physiological validity. ET data were recorded monocularly using a Pupil Labs device (Berlin, Germany) at 25Hz. The dataset description does not report explicit illumination control during recording^26^. Of the 40 recruited participants, ET data were available for 19. Four participants were excluded because fewer than 20 valid five-second stress windows could be extracted due to insufficient signal quality, resulting in a final sample of 15 participants. Because no eye tracker calibration was performed, gaze-based measures could not be derived. Consequently, the analysis was restricted to PD, in contrast to the calibrated binocular data available in the VR goalkeeper dataset. Table 3 summarizes the main characteristics of both datasets.

**Table 3:** Comparison of the virtual reality (VR) goalkeeper dataset and the ForDigitStress dataset^26^.

Feature	VR goalkeeper	ForDigitStress
Stressor	Serial recall task, performance feedback, and competition	Virtual job interview
Labeling method	Phase-based	Continuous
ET device	Tobii Pro	Pupil Labs
ET type	Binocular	Monocular
Sampling rate	90Hz	25Hz
Calibration	Five-point	None
Participants (analyzed / recruited)	27 / 30$^{*}$	15 / 40$^{\dagger }$
Extracted windows (non–stress / stress)	540 / 540	5,653 / 693

$^{*}$Three participants were excluded due to eye-tracking (ET) recording failures during data acquisition. $^{\dagger }$ ET data were available for 19 participants; four were excluded because fewer than 20 valid five-second stress windows could be extracted due to insufficient signal quality.

Given the differences between the datasets in sampling rate, eye tracker calibration procedures, and available ET signals, the preprocessing framework developed for the VR goalkeeper dataset was applied where possible and adapted to the ForDigitStress recordings as needed. Heimerl *et al.*^26^ provide stress annotations at the level of individual interview segments. In contrast, physiological validation relied on salivary cortisol, which reflects activation of the HPA axis and exhibits a delayed response, typically peaking 20min after stress exposure^2,47^. To ensure a clearer physiological separation between classes, we therefore used annotated interview segments as stress samples, while we extracted non-stress windows from the post-interview phase. During this phase, cortisol levels and perceived stress decline toward resting levels^26^, making it a more reliable low-stress reference than interview segments, which may still reflect anticipatory or residual stress responses.

Because the dataset provides only monocular PD, we could not average across eyes, and the values are not calibrated to physical units. Consequently, applying scale-dependent filters used for the VR goalkeeper dataset (e.g., out-of-bounds removal or dilation-speed-based artifact detection) would have required introducing arbitrary thresholds. To avoid this, we did not apply these filters. Instead, we relied on confidence-based handling of low-quality samples and segments using the tracker-provided confidence metric, which reflects pupil detection quality. We segmented PD signals into five-second windows, and assessed segment quality based on the Pupil Labs confidence metric (low quality: $< {80}\%$ of samples with confidence > 0.8). For windows not meeting this criterion, we shifted the window by ${20}\%$ of its length (one second) to obtain a usable segment; otherwise, we applied a ${50}\%$ overlap. We realigned windows containing changes in stress annotation to the most recent label transition. After preprocessing and quality filtering, the final dataset comprised 5,653 non-stress and 693 stress windows (Table 3).

**Fig. 3:**
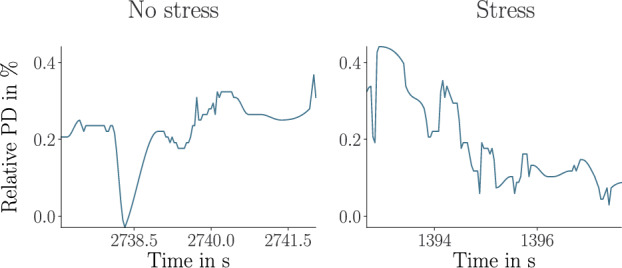
Representative preprocessed pupil diameter (PD) signal from the ForDigitStress dataset^26^, showing stress and non-stress windows used for model training.

After segmentation and window selection, invalid samples within the retained windows were identified using the same confidence threshold. To account for uncertainty around low-confidence detections, invalid regions were extended by marking the two adjacent samples on either side as invalid. These missing or unreliable samples were then interpolated using piecewise cubic Hermite interpolation, which preserves local signal shape while avoiding overshooting artifacts. Following interpolation, we applied divisive baseline correction (Equation 2) using a one-second artifact-free segment from the post-interview phase, selected after the initial 30s when tracker confidence consistently exceeded 0.8. Because no eye tracker calibration was performed, gaze-based measures such as visual angle, velocity, or fixations could not be computed. Figure 3 shows one representative preprocessed PD signal for each condition, stress and non-stress.

### Model training for the ForDigitStress dataset

Model training followed the same procedure and architectures as used for the VR goalkeeper dataset. Because the dataset is imbalanced, with substantially fewer stress than non-stress samples, adaptive synthetic sampling (ADASYN)^48^ was used to generate synthetic stress samples. ADASYN focuses sample generation on regions with sparse minority density, improving classification of difficult cases. Oversampling was applied only to training data within the nested subject-wise LOSO-CV: in the training portion of each inner fold and again when retraining the final model on the outer training set, while validation and test data remained unchanged.

Performance was evaluated using the macro-averaged F1-score ($F_{1,\text {macro}}$) across all LOSO-CV folds, giving equal weight to stress and non-stress classes (Equation 1). We additionally report the weighted F1-score ($F_{1,\text {weighted}}$), which accounts for class frequencies: $F_{1,\text {weighted}} = \frac{\sum _{i=1}^{C} w_i \cdot F_{1,i}}{\sum _{i=1}^{C} w_i}$, where $w_i$ denotes the number of instances in class *i* and $F_{1,i}$ the class-specific F1-score.

### Temporal attribution analysis

To improve interpretability and analyze which temporal regions contributed most strongly to model predictions, we performed post-hoc temporal attribution analysis on trained outer-fold models using held-out test samples. Temporal relevance was assessed using occlusion sensitivity analysis ^49^ by systematically masking local temporal windows and measuring the resulting decrease in predicted class score. Attribution profiles were aggregated across samples to obtain representative temporal relevance patterns. Additional attribution analyses, including gradient-based saliency maps and individual attribution examples, are provided in the Supplementary Section E.

## Results

Table 4 summarizes the RF results on the VR goalkeeper dataset. PD-based features achieved an F1-score of ${76.47}\%$, fixation features ${78.46}\%$, and the combined feature set performed best at ${83.64}\%$, with approximately eight features selected on average. A detailed analysis of feature-selection frequencies for the combined feature set is provided in Supplementary Fig. S2, offering additional insight into the relative importance and stability of individual features across LOSO-CV folds.

**Table 4:** Feature-based stress classification results on the virtual reality (VR) goalkeeper dataset^7^ using a random forest (RF) classifier. Values represent mean ± standard deviation across LOSO-CV folds to reflect variability across participants. Best results are shown in bold.

Feature subset	Accuracy	F1-score	# features
Pupil diameter (PD) statistics	76.85 ± 9.24	76.47 ± 10.79	5.37 ± 1.97
Fixation characteristics	76.39 ± 9.99	78.46 ± 8.85	2.30 ± 0.46
**Combined**	**83.70 ± 9.44**	**83.64 ± 10.06**	**8.07 ± 0.98**

For time-series-based classification on the VR goalkeeper dataset (Table 5), the best performance was achieved by the three-layer ConvLSTM using the asymptotic-model input (F1 = ${95.98}\%$). Velocity, acceleration, visual angle, and asymptotic-model inputs all achieved F1-scores above ${94}\%$ for at least one architecture. For PD, the CNN achieved the highest performance (F1 = ${88.85}\%$).

Inferential statistical comparisons between the best-performing DL models and the corresponding feature-based baselines showed significant differences after Holm correction (all adjusted $p <.001$; Supplementary Section D.2). Complementary subject-level error analysis (Supplementary Section F.1) showed that a subset of participants consistently yielded lower macro F1-scores across multiple model architectures and input signals. Corresponding confidence intervals, receiver operating characteristic (ROC) and precision–recall (PR) analyses, and additional error analyses are provided in the Supplementary Materials.

**Fig. 4:**
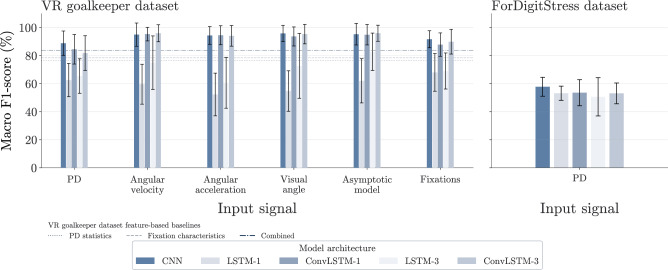
Macro F1-score comparison across model architectures for both datasets. Left: virtual reality (VR) goalkeeper dataset across input signals and model architectures. Right: ForDigitStress dataset using pupil diameter (PD) as input. Error bars indicate standard deviation across outer leave-one-subject-out cross-validation (LOSO-CV) folds. Horizontal reference lines in the VR goalkeeper panel indicate feature-based random forest (RF) baseline performance.

Performance on the ForDigitStress dataset (Table 6 and Fig. 4) was lower overall. The CNN achieved the highest macro F1-score (${57.77}\%$), while the single-layer LSTM achieved the highest weighted F1-score (${80.89}\%$). The larger weighted F1-scores reflect the pronounced class imbalance of the dataset (Table 3), in which non-stress samples substantially outnumber stress samples. Corresponding ROC and PR curves are provided in Supplementary Fig. S6.

**Table 5:** Time-series-based stress classification results on the virtual reality (VR) goalkeeper dataset^7^. Values represent mean ± standard deviation F1-scores (%) across LOSO-CV folds. LSTM-1 and ConvLSTM-1 denote one-layer variants, while LSTM-3 and ConvLSTM-3 denote three-layer variants of the respective architectures. Best overall and best pupil diameter (PD) results are shown in bold.

Input signal	CNN	LSTM-1	ConvLSTM-1	LSTM-3	ConvLSTM-3
PD	**88.85 ± 8.70**	62.53 ± 11.84	84.51 ± 10.53	65.39 ± 12.34	81.79 ± 12.36
Velocity	94.99 ± 8.28	59.61 ± 13.41	95.33 ± 4.84	74.97 ± 19.09	95.96 ± 6.07
Acceleration	94.36 ± 6.33	52.28 ± 15.23	94.55 ± 6.77	60.56 ± 18.14	94.12 ± 7.35
Visual angle	95.81 ± 5.84	54.72 ± 14.45	93.67 ± 6.80	72.67 ± 23.16	95.33 ± 6.91
Asymptotic model	95.26 ± 7.67	61.99 ± 15.76	94.91 ± 7.28	82.73 ± 13.33	**95.98 ± 5.72**
Fixations	91.67 ± 6.04	68.01 ± 13.45	87.84 ± 8.33	69.02 ± 12.83	89.92 ± 8.83

**Table 6:** Time-series-based stress classification results on the ForDigitStress dataset^26^ using pupil diameter (PD). Values represent mean ± standard deviation F1-scores (%) across LOSO-CV folds. LSTM-1 and ConvLSTM-1 denote one-layer variants, while LSTM-3 and ConvLSTM-3 denote three-layer variants of the respective architectures. Best results are shown in bold.

	CNN	LSTM-1	ConvLSTM-1	LSTM-3	ConvLSTM-3
$F1_{macro}$	**57.77 ± 6.72**	53.13 ± 5.06	53.54 ± 9.29	50.60 ± 13.66	53.06 ± 7.44
$F1_{weighted}$	79.57 ± 7.14	**80.89 ± 5.47**	75.79 ± 14.16	73.28 ± 18.29	80.44 ± 6.93

**Fig. 5:**
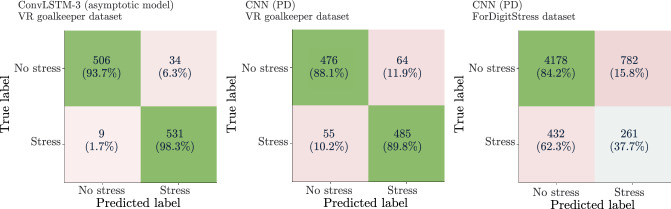
Aggregated confusion matrices for representative models. Left: best-performing LSTM, and ConvLSTM-3 model on the virtual reality (VR) goalkeeper dataset (asymptotic-model input). Middle: best pupil diameter (PD)-based CNN on the VR goalkeeper dataset. Right: best PD-based CNN on the ForDigitStress dataset. Values represent absolute counts with row-normalized percentages in parentheses.

To provide additional insight into class-specific prediction behavior, Fig. 5 shows aggregated confusion matrices for representative models: the best-performing overall model (ConvLSTM-3 with asymptotic-model input) on the VR goalkeeper dataset, the best PD-based model (CNN) on the same dataset, and the best PD-based model on the ForDigitStress dataset (CNN). Confusion matrices were aggregated across all outer folds of the nested LOSO-CV procedure. The models on the VR goalkeeper dataset correctly classified most stress and non-stress samples, whereas the ForDigitStress model showed a larger number of stress samples misclassified as non-stress, resulting in lower recall for the stress class.

To investigate whether the reduced performance on the ForDigitStress dataset was primarily caused by technical recording differences, we conducted additional robustness experiments on the VR goalkeeper dataset by progressively approximating characteristics of the ForDigitStress recordings, including reduced sampling rate (25Hz), monocular pupil recordings, and the removal of scale-dependent pupil filtering steps. For these experiments, the CNN architecture was selected because it achieved the highest macro F1-score for PD for both datasets. As summarized in Table 7, reducing the sampling rate from 90Hz to 25Hz, using monocular instead of binocular pupil signals, and removing scale-dependent filtering steps resulted in macro F1-scores between ${86.96}\%$ and ${88.85}\%$. Across all robustness conditions, performance decreased by less than two percentage points relative to the baseline configuration.

**Table 7:** Robustness experiments investigating the influence of sampling rate and preprocessing differences on pupil diameter (PD)–based stress classification. Starting from the fully preprocessed binocular PD signal used in the main analysis (baseline), preprocessing was progressively simplified to approximate characteristics of the ForDigitStress dataset. Performance is reported as macro F1-score (mean ± standard deviation) across LOSO-CV folds.

Condition	Sampling rate	PD	Filter	F1-score (%)
Baseline	90 Hz	bino. mean	yes	$88.85 \pm 8.70$
Downsampled	25 Hz	bino. mean	yes	$87.24 \pm 9.10$
Monocular	25 Hz	right eye	yes	$87.42 \pm 9.60$
Monocular + no filter	25 Hz	right eye	no	$86.96 \pm 9.32$

**Fig. 6:**
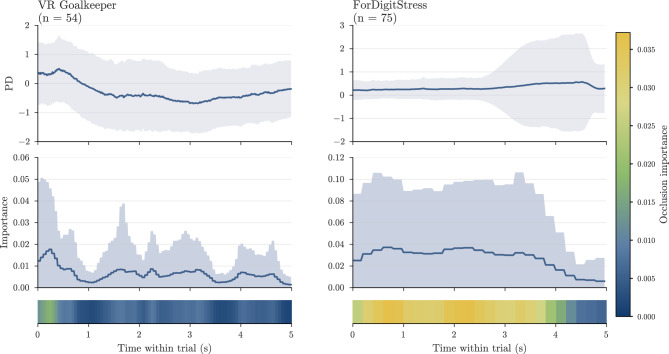
Comparison of aggregated temporal occlusion profiles for correctly classified stress samples using the best-performing pupil diameter (PD)-based convolutional neural network (CNN) models on the virtual reality (VR) goalkeeper and ForDigitStress datasets. The upper row shows the mean normalized PD signal across samples, with shaded regions indicating ± one standard deviation. The middle row shows the corresponding mean occlusion-importance profiles, and the lower row provides a color-coded representation of the same temporal relevance patterns. The VR goalkeeper dataset exhibits more localized early relevance peaks, whereas temporal relevance in the ForDigitStress dataset is more broadly distributed across the analyzed window.

To further investigate temporal differences in model behavior across datasets, Fig. 6 compares aggregated temporal occlusion profiles for correctly classified stress samples using the best-performing PD-based CNN models on both datasets. The VR goalkeeper dataset exhibits more localized temporal relevance with pronounced early peaks, whereas the ForDigitStress dataset shows more broadly distributed relevance across the analyzed window.

Additional analyses and implementation details are provided in the Supplementary Materials. These include detailed confidence interval analysis across outer folds (Section D.1), extended statistical comparisons and paired fold-wise difference visualizations (Section D.2), ROC and PR analyses for both datasets (Section D.3), and computational complexity analysis (Section D.4), which revealed substantial differences in parameter counts and inference costs across architectures. Additional attribution analyses are provided in Section E, while subject-level, noise-related, and temporal error analyses are reported in Section F. Furthermore, additional implementation details for the robustness experiments are provided in Section G.

## Discussion

This study investigated whether neural networks applied to raw ET time series can improve task-agnostic stress classification and which ET signals are most informative. The task-agnostic feature-based baseline approach achieved an accuracy of ${83.70}\%$, slightly below previously reported task-specific features on the same dataset^7^. This difference is likely due to the omission of task-specific information in the present approach, as context-dependent gaze behavior provides additional discriminative cues that enhance classification performance. A more detailed view is provided by the feature-wise analysis. Using only PD statistics resulted in an F1-score of ${76.47}\%$, while fixation features achieved

The time-series-based DL models clearly outperformed the feature-based results discussed above, achieving a macro F1-score of ${95.98}\pm{5.72}\%$ compared to ${83.64}\pm{10.06}\%$ for the feature-based baseline. Both values represent averages across subject-wise LOSO-CV folds. In addition to the higher mean performance, the lower standard deviation for the DL models indicates more consistent performance across participants. This is further supported by the corresponding confidence intervals (Supplementary Section D.1), suggesting that the observed improvements are consistently observed across participants. Consistent with prior work^23,25^, this finding highlights the advantage of learning directly from raw ET signals.

Few studies directly compare feature-based and DL approaches for ET-based stress detection. The achieved performance lies at the upper end of results reported in prior unimodal ET studies, which typically reach accuracies from ${70}\%$ to ${90}\%$^21,22,24,26,50^. Notably, our results were obtained under moderate visuomotor activity, whereas most existing work focuses on sedentary settings^18,22,50^. This suggests that the proposed DL approach remains effective in less controlled, more dynamic scenarios although its robustness is expected to depend on recording conditions and dataset characteristics. However, direct comparisons across studies remain limited, as highlighted in Table 1, due to substantial differences in stress paradigms, sensor setups, and dataset characteristics. While our findings demonstrate feasibility in the VR goalkeeper setting, they are constrained by the dataset’s size and homogeneity and therefore cannot be readily generalized to broader unconstrained movement scenarios. Nevertheless, prior work indicates that reliable ET recordings are achievable even under substantial movement^51^, supporting the feasibility of stress detection in dynamic environments.

Analysis of individual input signals revealed clear interactions between signal characteristics and model architecture. Overall, dynamic gaze signals achieved the highest performance, with several configurations exceeding ${95}\%$ on the VR goalkeeper dataset (Table 5). These signals, including velocity, visual angle, and asymptotic-model representations, exhibit rich temporal dynamics with both local and sequential structure. The ConvLSTM-3 model achieved the best performance for these inputs, consistent with its ability to combine convolutional feature extraction with temporal modeling^45^. At the same time, CNNs also performed strongly on these signals, with results approaching those of the best ConvLSTM-3 configurations and substantially exceeding performance for more static inputs. ROC and PR analyses (Supplementary Fig. S5) indicate only minor differences between the best-performing architectures. In contrast, for less dynamically varying representations such as PD and fixation statistics, CNNs consistently outperformed other architectures, suggesting that these inputs contain less complex temporal structure and can be effectively modeled using local temporal patterns. LSTMs showed lower performance overall, likely because the fixed-length windows used in this study limit the temporal continuity required by purely recurrent architectures.

Within this group of more static signals, PD is of particular interest, as it reflects autonomic processes and is fundamentally different from gaze-based measures. In addition, PD was the only signal available in both datasets, providing a direct point of comparison across experimental conditions. The PD-based CNN improved the F1-score by nearly nine percentage points over the baseline RF using PD statistics, demonstrating the advantage of DL for task-agnostic physiological signals. This aligns with prior work identifying PD as a strong autonomic stress marker^17,52^.

While performance on the VR goalkeeper dataset was strong (CNN: ${88.85}\%$ macro F1-score), it decreased substantially on the ForDigitStress dataset (CNN: ${57.77}\%$ macro F1-score, LSTM-1: ${80.89}\%$ weighted F1-score), highlighting that model performance is fundamentally constrained by dataset characteristics. To place these results in context, a direct comparison with the original ForDigitStress study is only possible to a limited extent. The original work reports an accuracy of approximately ${70.2}\%$, but differences in evaluation metrics (e.g., accuracy vs. macro or weighted F1-score) complicate direct comparison. A key factor underlying these differences is the temporal validity of stress labels. Physiological validation of stress responses, for example using salivary markers, typically operates at the level of experimental phases due to delayed response dynamics and therefore does not provide precise ground truth at the level of individual time windows or questions. As a result, labels in the ForDigitStress dataset, as in many comparable studies, are derived from stress-inducing task conditions or annotated segments and therefore only approximate underlying stress states. In this study, we address this limitation through a modified labeling strategy: stress windows are extracted from annotated interview segments following the original protocol, whereas non-stress samples are drawn from the post-interview phase to better reflect physiological recovery. While this improves physiological plausibility, it reduces comparability with prior results based on within-interview annotations. Consequently, differences in performance between studies cannot be attributed solely to model choice, but also reflect differences in labeling strategy and the evaluation protocol. More generally, this limitation becomes particularly relevant for finer-grained stress detection. As discussed in the literature, condition-based labeling does not necessarily reflect individual and time-varying stress responses^53^. Future work should therefore consider combining physiological signals with subjective and contextual information to obtain a more comprehensive view of stress. However, these sources should be regarded as complementary proxies rather than definitive ground truth.

Beyond differences in label validity, performance variations can also be attributed to model behavior and dataset-specific characteristics. To better understand these effects relative to the VR goalkeeper dataset, we analyze model performance within the ForDigitStress dataset across multiple complementary perspectives. At the model level, differences in class-specific performance become apparent. While the CNN achieves the highest macro F1-score, the LSTM-1 model yields the best weighted F1-score, indicating differences in how models balance class-specific performance. Consistent with this, combined ROC and PR analysis (Supplementary Fig. S6) reveals reduced and threshold-dependent class separability. ROC curves for different architectures intersect, showing that model ranking depends on the chosen operating point: CNNs achieve higher performance at low false positive rates, whereas recurrent architectures provide higher recall at more permissive thresholds. The corresponding PR curves further highlight the difficulty of maintaining high precision at increasing recall, consistent with the pronounced class asymmetry observed in the confusion matrices (Fig. 5), where recall is substantially lower for the stress class. Subject-level error analysis (Supplementary Section F.1) indicates participant-specific differences in classification difficulty.

To assess the role of technical recording factors, we conducted additional robustness experiments for the VR goalkeeper dataset (Table 7) by progressively approximating the recording characteristics of the ForDigitStress dataset, including lower sampling rate, monocular pupil signals, and the removal of calibration-dependent filtering steps. These modifications resulted in only minor performance changes for pupil-based classification, indicating that individual technical factors alone do not account for the observed differences. Additional analysis of PD signal quality (Supplementary Section F.2) further supports this interpretation: while noise-related factors are associated with reduced performance in the ForDigitStress dataset, their effect is weak and inconsistent in the VR dataset, suggesting that signal quality contributes to performance variability but does not fully explain the observed differences. Importantly, this robustness analysis is limited to PD signals, for which comparable preprocessing approximations could be defined. For gaze-derived signals, such as velocity and acceleration, no equivalent cross-dataset approximation was performed. These signals rely on temporal differentiation and are therefore more sensitive to temporal resolution and noise. Because saccades typically last only 20ms to 50ms, low sampling rates may undersample rapid eye movements and reduce the stability of such features^38,54^. Consequently, while sampling rate showed only minor effects for PD, its influence on dynamic gaze features is expected to be more pronounced due to their reliance on high-frequency temporal information. Artificially reducing sampling rate or introducing noise would therefore not only approximate recording conditions, but may also substantially distort the underlying signal characteristics, limiting the interpretability of such analyses.

Beyond technical recording factors, the temporal structure of the signals provides a further perspective on the observed dataset differences. As illustrated by the aggregated temporal occlusion profiles in Fig. 6, informative signal components in the VR goalkeeper dataset are primarily concentrated in the early part of the segment, consistent with rapid, event-related autonomic responses such as transient pupil dilation following salient stimuli^12,55^. These phasic responses are typically short-lived and time-locked to stimulus onset, which explains the localized attribution patterns. In contrast, the more distributed attribution patterns observed in the ForDigitStress dataset indicate that the CNN relies on informative features across a broader portion of the input segment, rather than on sharply localized patterns. The figure summarizes representative attribution patterns for correctly classified stress samples using the best-performing PD-based CNN models, while additional attribution examples, saliency-based analyses, and architecture comparisons are provided in Supplementary Section E. This is in line with more subtle, psychosocial stress responses, which are characterized by sustained, tonic increases in arousal and more gradual temporal dynamics, reflected in slowly evolving PD and reduced variability in gaze behavior. At the same time, the reduced attribution toward the end of the five-second windows suggests that, from the model’s perspective, sufficient discriminative information is often already captured in earlier parts of the window, while the remaining portion contributes less. This implies that relevant signal characteristics are distributed across the segment but are not required uniformly, which is consistent with temporally extended but weakly structured stress responses in psychosocial contexts^2,47,56^. Together, these findings indicate that attribution patterns reflect differences in the temporal dynamics of stress responses and highlight that model design should be adapted to the underlying stress characteristics of the task. This difference in temporal structure may also contribute to the observed performance differences. Models that rely on fixed receptive fields or predominantly local temporal patterns may be less suited to capturing more distributed or weakly structured signal characteristics. Architectures designed to model temporal dependencies across multiple receptive-field sizes, such as temporal convolutional networks^57^, may therefore provide advantages in such settings, as differences in the ability of DL architectures to capture temporal dependencies at multiple scales have been identified as a key factor in time-series modeling^58^. Similarly, attention-based architectures may be beneficial in more heterogeneous scenarios, as transformer-based models can integrate information across longer temporal ranges^59^. However, such architectures were not considered in the present study, as the primary objective was to systematically investigate the interaction between ET signal characteristics, dataset properties, and established DL architectures under heterogeneous recording conditions. In addition, their applicability to relatively small and noisy ET datasets remains an open question for future work, especially given their increased model complexity and potential sensitivity to limited and noisy data.

Taken together, these analyses provide a consistent picture of the factors driving the observed performance differences. Controlled robustness experiments indicate that technical factors, such as sampling rate or preprocessing differences, have only a limited impact and are therefore unlikely to be the primary drivers in isolation. Instead, the remaining dataset-level differences are more likely to dominate performance. In particular, reduced calibration quality, differences in task design and stress induction, increased class imbalance, and the smaller number of subjects in the ForDigitStress dataset directly affect signal stability, class separability, and the consistency of stress-related responses. Although their relative contributions cannot be quantified, the results indicate that performance degradation arises from their combined effect rather than isolated technical limitations. Importantly, this is not merely a limitation but a central finding: unimodal ET-based stress detection is highly sensitive to calibration quality, task structure, and dataset composition.

### Limitations and practical considerations

Our findings should be interpreted in light of several limitations. ET signals may be influenced by confounding factors such as illumination variation, fatigue, emotional state, task difficulty, and inter-individual differences in baseline psychological traits^12^. In retrospective datasets, these influences are typically not explicitly measured and therefore cannot be disentangled from stress-related responses post hoc. In the present study, several of these factors were mitigated through the experimental design, including controlled illumination in the VR goalkeeper dataset, relatively short recording sessions, and subject-wise cross-validation. Nevertheless, participant-specific variability in classification performance (Supplementary Section F.1) indicates that inter-individual differences remain a relevant factor. In addition, the analysis of error rates across sample order (Supplementary Section F.3) showed no systematic increase over time, suggesting that fatigue-related effects are unlikely to be a dominant confound. Instead, the elevated error rate at the beginning of the task points to a potential initial adaptation or transition effect, which may reflect baseline-related differences in how participants enter the stress condition. Prior work on pupil-based workload estimation has shown that separating workload-related pupil changes from illumination effects requires tightly controlled lighting conditions and dedicated modeling approaches under fixed task settings^60^. This highlights the importance of accounting for such confounding factors during data acquisition. For practical deployment, where these influences are typically uncontrolled, future work should therefore focus on recording protocols that explicitly capture relevant contextual variables or allow their systematic variation, enabling more robust and interpretable stress detection under naturalistic conditions.

Another limitation is the sample size and homogeneity of the datasets (27 and 15 participants), which may restrict generalizability, although they are comparable to existing ET-based stress studies (6–44 participants^17–19,21–25^). Given the sample sizes and the subject-wise cross-validation design, results are primarily interpreted descriptively, and formal statistical comparisons should be treated with caution. In this context, the present work should be understood as a proof-of-concept demonstrating the feasibility of the proposed approach under semi-controlled and context-dependent conditions.

In addition, both datasets exhibit limited demographic diversity, with a strong bias toward young adults and, in the VR goalkeeper dataset, a predominance of male participants. Such relatively homogeneous study populations prevent meaningful subgroup analysis and limit conclusions regarding fairness and generalization across populations. This is particularly relevant given known age-related differences in pupil size and responsiveness, suggesting that models may not directly transfer to other age groups without adaptation^61^.

These limitations are not specific to the present study but reflect a broader challenge in the field. While individual datasets are typically small and demographically homogeneous, they differ substantially in recording conditions, stress induction paradigms, and annotation strategies^26,28^. Within these constraints, the present work focuses on within-dataset generalization under strict subject-wise separation. Accordingly, the comparison across datasets should be interpreted as a cross-context analysis that highlights the sensitivity of unimodal ET-based stress classification to such variations, rather than as formal external validation on an independent test cohort collected under comparable and harmonized conditions. Enabling robust cross-dataset generalization and fairness-aware evaluation will therefore require larger, more diverse, and systematically collected cohorts across multiple sites and application contexts, as well as validation on independent cohorts under harmonized protocols.

From an application perspective, the results suggest that unimodal ET-based stress detection is most suitable for semi-controlled settings with stable recording conditions and well-defined task structures. Such conditions are typically found in structured interaction contexts, for example in VR-based training systems, workplace assessments, or controlled human–computer interaction scenarios, where task context and recording quality can be managed. Within such settings, the proposed pipeline supports short-window inference using five-second segments. In line with the positioning of unimodal ET as a low-burden approach, this design prioritizes scalability and reduced hardware requirements, which are key advantages for practical deployment. A post hoc complexity analysis (Supplementary Table S9) shows that the CNN model is the least computationally demanding architecture, with substantially fewer parameters (${0.25}\pm{0.21}$ million) than more complex models such as ConvLSTM-3 (${3.60}\pm{4.03}$ million). Despite this difference in complexity, classification performance for the asymptotic-model input is comparable across architectures (Table 5), indicating that increased model complexity does not necessarily yield proportionally higher performance. This suggests that, for real-time or near-real-time applications such as stress-aware human–computer interaction or VR-based training systems, simpler architectures may offer a favorable trade-off between performance and computational requirements. Beyond computational considerations, application-specific decision requirements must also be taken into account. The threshold-dependent behavior observed in ROC and PR analysis suggests that different architectures may be preferable depending on the intended system behavior and its tolerance to errors. In continuous monitoring or feedback-oriented applications, the goal is to detect stress responses reliably over time. In this case, higher recall may be desirable to avoid missing relevant stress events, even if this leads to occasional false alarms. In contrast, in adaptive systems that actively modify the user experience—such as VR-based training systems that adjust task difficulty or provide real-time feedback—incorrect interventions can disrupt the interaction. Here, false positives become more critical, as unnecessary adaptations—such as reducing task difficulty—may degrade the user experience. In such settings, a more conservative decision threshold may therefore be preferable. In addition, while the present study focuses on classification performance, predicted probabilities were not explicitly calibrated and therefore cannot be interpreted as reliable confidence estimates. For practical use, post hoc calibration methods (e.g. temperature scaling^62^) would be required to support uncertainty-aware decision-making, such as threshold adjustment or abstention in uncertain cases^63^. For example, in a VR-based training scenario, a system could require a higher confidence threshold before adapting task difficulty and abstain from reducing task complexity when predictions are uncertain, thereby avoiding inappropriate adjustments that could disrupt the intended training progression. Incorporating calibrated uncertainty estimates thus represents an important direction for future work, particularly in user-facing or safety-critical settings. Finally, practical deployment depends not only on model characteristics but also on sensor capabilities and operating conditions. Some model configurations rely on calibrated gaze-direction measurements that may not be available in everyday environments. In addition, long-term deployment introduces challenges such as calibration drift and missing samples caused by blinks or headset slippage^33,38^. However, recent work shows that calibration drift can be mitigated through algorithmic correction and continual calibration strategies, reducing the need for explicit user-driven recalibration^64,65^.

Ethical considerations are also relevant for the practical deployment of ET-based stress detection. Inferring stress or related affective states from physiological and behavioral signals remains inherently uncertain, and similar approaches in emotion recognition have been shown to be susceptible to misclassification and systematic bias. If such errors or biases occur, they may lead to incorrect or unfair assessments, particularly in sensitive contexts. For example, a system that incorrectly infers elevated stress could trigger inappropriate feedback or interventions, or misrepresent a user’s state in a way that influences how they are evaluated or treated. These risks are especially critical in high-stakes application scenarios, such as workplace monitoring or screening tools, where automated stress inference could directly influence decisions about individuals. Such uses raise concerns about fairness, autonomy, and the potential misuse of inferred internal states. These challenges have motivated increasing calls for regulatory oversight of systems that infer internal states from observable data^66^. From a regulatory perspective, such applications are addressed by emerging frameworks such as the European Union’s Artificial Intelligence Act, which classifies certain uses of biometric data and emotion inference systems as high-risk or prohibited, particularly in sensitive domains such as employment or education, and introduces requirements for transparency, risk management, and human oversight. At the same time, these regulatory frameworks provide an important foundation for the responsible development of such systems. When designed and deployed in alignment with these guidelines, for example in user-centered applications such as adaptive human–computer interaction or VR-based training, ET-based stress detection may support real-time feedback or task adaptation without directly affecting high-stakes decisions. Rather than restricting innovation, such regulations can guide the safe, ethical, and context-appropriate use of these technologies.

## Conclusion

This work investigated whether unimodal ET time series support task-agnostic stress detection beyond static laboratory tasks. The results show that ET signals alone contain informative patterns related to stress-associated autonomic and oculomotor responses and that these can be effectively captured from raw time-series data. In particular, the findings indicate that model performance depends on the alignment between signal characteristics and model architecture, with more dynamic signals benefiting from architectures that explicitly model temporal dependencies. Together, this highlights the potential of unimodal ET as a low-burden, scalable approach to stress detection. At the same time, performance decreased substantially on the ForDigitStress dataset, reflecting differences in recording conditions and dataset characteristics. Additional analysis shows that this gap cannot be explained by isolated technical factors such as sampling rate or preprocessing alone. Instead, effective learning depends on the combined influence of data integrity, calibration quality, signal properties, and task design, which jointly determine the separability and stability of stress-related patterns. Finally, as the results are based on cross-context comparisons across datasets with differing characteristics, they do not constitute formal external validation and, therefore, do not demonstrate robust generalization to unconstrained conditions. Achieving deployment under unconstrained conditions will require improved recording quality, reliable calibration, larger and more diverse datasets, and validation on independent cohorts collected under harmonized protocols. Overall, the results indicate that unimodal ET-based stress detection is a promising alternative to more complex multimodal systems, but its effectiveness is fundamentally shaped by data quality, calibration, and task design, ultimately determining both its current limitations and its potential for deployment beyond controlled environments.

## Supplementary Information

Supplementary Information.

## Data Availability

Data availability The VR Goalkeeper dataset generated during the current study is publicly available via Zenodo at https://zenodo.org/records/17972964. The ForDigitStress dataset analysed during the current study is available from the dataset provider for research and non-commercial use, subject to access approval and the dataset EULA. Access can be requested at https://hcai.eu/fordigitstress. Because ForDigitStress is a third-party dataset with access restrictions, it is not redistributed by the authors or included in the code repository. Processed VR Goalkeeper dataframes and compact source-data/result files supporting reproduction of the reported VR Goalkeeper analyses are provided in the associated code repository. Code availability The complete code repository used for preprocessing, feature extraction, model training, evaluation, robustness analyses, visualization, and supplementary analyses is publicly available at https://github.com/maikestoe/et-based-stress-classification. The repository additionally includes experiment configurations, representative experiment logs, source-data/result files, and documentation supporting reproduction of the analyses presented in the manuscript and Supplementary Materials.

## Code availability

The complete code repository used for preprocessing, feature extraction, model training, evaluation, robustness analyses, visualization, and supplementary analyses is publicly available at https://github.com/maikestoe/et-based-stress-classification. The repository additionally includes experiment configurations, representative experiment logs, source-data/result files, and documentation supporting reproduction of the analyses presented in the manuscript and Supplementary Materials.
