# Supplementary material for: Classifying mental stress from eye tracking data: deep learning approaches for out-of-the-lab conditions
Source: Sci Rep. 2026 Jun 20;16:19188. doi: 10.1038/s41598-026-58429-7 (PMC13283211; doi:10.1038/s41598-026-58429-7)
Supplement: Supplementary file 1 — Supplementary Information. [file 41598_2026_58429_MOESM1_ESM.pdf]

Supplementary Materials for

Classifying Mental Stress from Eye Tracking Data: Deep Learning Approaches for Out-of-the-Lab Conditions

Maike Laut, Eva Dorschky, Robert Richer, Nicolas Rohleder, Bjoern M. Eskofier

This Supplementary Material provides additional methodological details, extended analyses, and supporting visualizations complementing the results presented in the main manuscript. Core findings and representative analyses are summarized in the manuscript, while the supplementary sections provide detailed statistical comparisons, additional attribution analyses, extended evaluation metrics, subject-level and noise-related error analyses, computational complexity analysis, and implementation details for the robustness experiments.

A Hyperparameter optimization ranges

Hyperparameter optimization was performed using Optuna[1].

A.1 Random forest models

Table S1. Hyperparameter and search space for the random forest (RF) models.

Hyperparameter	Sampling	Search Space
n estimators	fixed	300
max features	categorical	$\sqrt{\text{max features}}$
max depth	categorical	$\in \{10, 157, 305, 452, 600\}$
min samples split	categorical	$\in \{2, 4, 6\}$
min samples leaf	categorical	$\in \{1, 2, 4\}$
bootstrap	fixed	True
n features	integer	$\in \{1, \ldots, 18\}$

A.2 Neural network training

Table S2. Hyperparameter optimization ranges for neural network training.

Hyperparameter	Sampling	Search Space
learning rate	log uniform distribution	$\in [1 \times 10^{-5}, 1 \times 10^{-2})$
alpha	floating point value	$\in [0.1, 1.0)$
gamma	floating point value	$\in [0, 5)$
batch size	fixed	32

A.3 CNN models

Table S3. Hyperparameter and search space for the convolutional neural network (CNN) models.

Hyperparameter	Sampling	Search Space
filter_1	categorical	$2^N, N \in \{6, \ldots, 9\}$
filter_2	categorical	$2^N, N \in \{4, \ldots, 8\}$
filter_3	categorical	$2^N, N \in \{4, \ldots, 8\}$
pooling size	categorical	$\in \{2, 3, 4, 5\}$
kernel size	categorical	$\in \{2, 3, 4\}$
dropout_1	floating point value	$\in [0.1, 0.5)$
dropout_2	floating point value	$\in [0.1, 0.5)$
dropout_3	floating point value	$\in [0.1, 0.5)$
units (dense)	categorical	$2^N, N \in \{4, \ldots, 7\}$

A.4 LSTM-1 models

Table S4. Hyperparameter and search space for the single layer long short-term memory (LSTM)-1 models.

Hyperparameter	Sampling	Search Space
units	categorical	$2^N, N \in \{6,\ldots,9\}$
L1 regularizer	log uniform distribution	$\in [1\times10^{-8}, 1\times10^{-1})$
L2 regularizer	log uniform distribution	$\in [1\times10^{-8}, 1\times10^{-1})$
clipvalue	log uniform distribution	$\in [1\times10^{-8}, 1)$

A.5 ConvLSTM-1 models

Table S5. Hyperparameter and search space for the single layer convolutional long short-term memory (ConvLSTM)-1 models.

Hyperparameter	Sampling	Search Space
filter	categorical	$2^N, N \in \{6,\ldots,9\}$
kernel size	categorical	$\in \{2,3,4\}$
dropout	floating point value	$\in \{0.1, 0.5\}$
number of segments	categorical	$\in \{5, 25\}$

A.6 LSTM-3 models

Table S6. Hyperparameter and search space for the three layer convolutional long short-term memory (ConvLSTM)-3 models.

Hyperparameter	Sampling	Search Space
units_1	categorical	$2^N, N \in \{4,\ldots,9\}$
units_2	categorical	$2^N, N \in \{4,\ldots,9\}$
units_3	categorical	$2^N, N \in \{4,\ldots,9\}$
L1 regularizer	log uniform distribution	$\in [1\times10^{-8}, 1\times10^{-1})$
L2 regularizer	log uniform distribution	$\in [1\times10^{-8}, 1\times10^{-1})$
clipvalue	log uniform distribution	$\in [1\times10^{-8}, 1)$

A.7 ConvLSTM-3 models

Table S7. Hyperparameter and search space for the three layer convolutional long short-term memory (ConvLSTM)-3 models.

Hyperparameter	Sampling	Search Space
filter_1	categorical	$2^N, N \in \{6,\ldots,9\}$
filter_2	categorical	$2^N, N \in \{6,\ldots,9\}$
filter_3	categorical	$2^N, N \in \{6,\ldots,9\}$
kernel size	categorical	$2^N, N \in \{4,\ldots,9\}$
dropout_1	floating point value	$\in [0.1, 0.5)$
dropout_2	floating point value	$\in [0.1, 0.5)$
number of segments	categorical	$\in \{5, 25\}$
units (dense)	categorical	$2^N, N \in \{6,\ldots,9\}$

B Training stability and learning behavior

To provide additional transparency regarding training stability and potential overfitting, we report loss curves from the final training runs of representative outer folds in the leave-one-subject-out cross-validation (LOSO-CV) procedure. The results are shown for the ConvLSTM-3 model with asymptotic-model input on the virtual reality (VR) goalkeeper dataset, which achieved the highest overall performance in the main experiments (F1-score: 95.98 ± 5.72). Figure S1 illustrates the evolution of training and validation loss across epochs for these folds. The reported loss corresponds to the categorical focal cross-entropy used during model training, with fold-specific α and γ parameters selected during hyperparameter optimization[2].

Model selection was based on the epoch achieving the highest validation macro F1-score, with the corresponding weights restored for final evaluation. While the loss curves show the full training process, the selected model therefore corresponds to an intermediate epoch and is not explicitly highlighted.

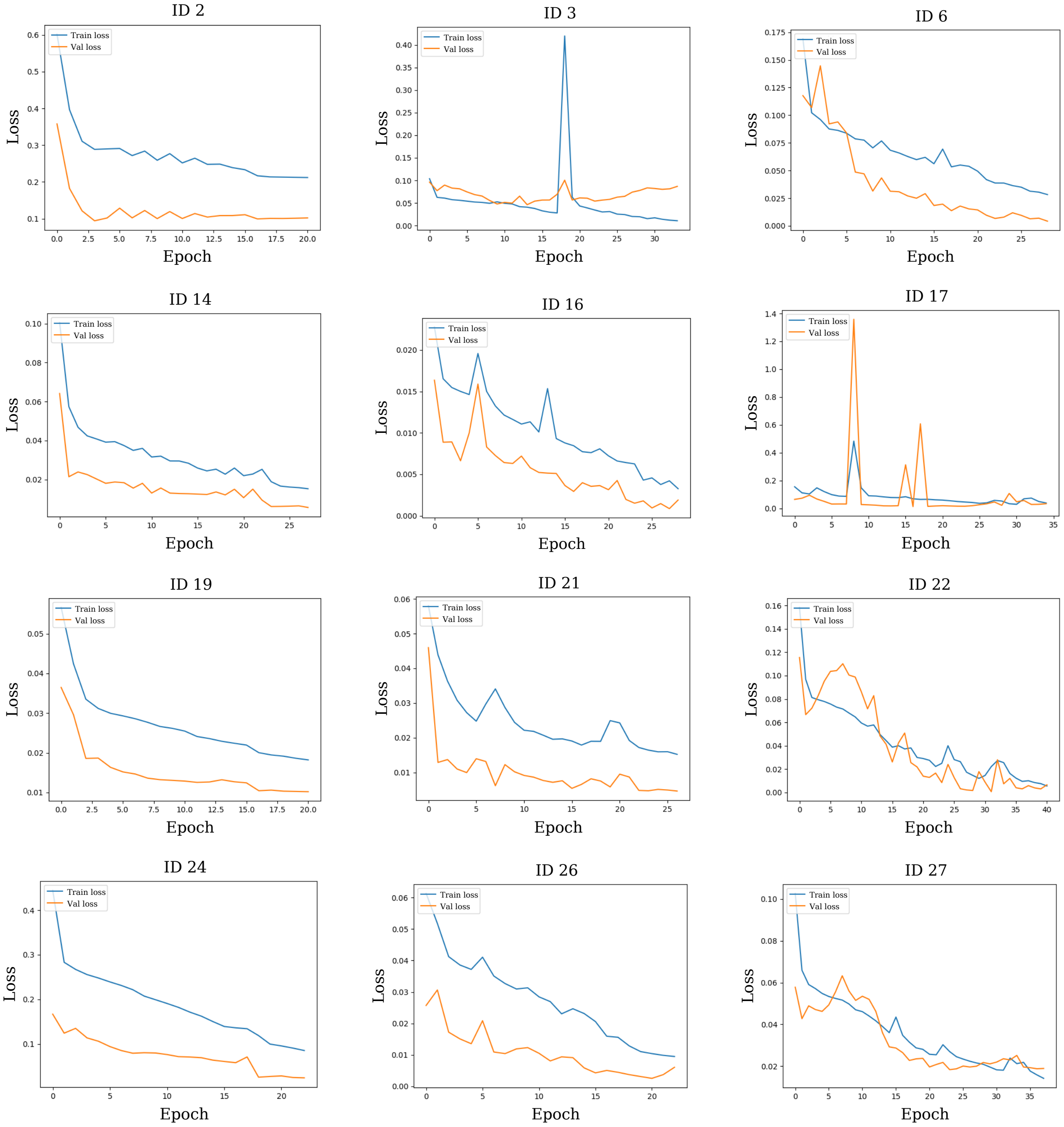

Figure S1. Training and validation categorical focal loss across epochs for selected outer folds (held-out participants) of the leave-one-subject-out cross-validation (LOSO-CV) procedure. Results are shown for the convolutional long short-term memory (ConvLSTM)-3 model with asymptotic-model input on the virtual reality (VR) goalkeeper dataset. Blue and orange curves indicate training and validation loss, respectively. Model selection was based on the epoch with the highest validation macro F1-score (not shown).

Across the selected folds, training loss generally decreased over epochs, indicating successful optimization of the models. Validation loss showed more fold-specific behavior: in most folds it remained low or decreased together with training loss, whereas some folds exhibited transient fluctuations or slight increases in later epochs. In several folds, validation loss was lower than training loss. This can occur because training loss is computed with training-time regularization active, particularly dropout, whereas validation loss is computed in inference mode with dropout disabled. In addition, the subject-wise LOSO-CV setup can lead to fold-specific differences in validation difficulty across held-out participants.

Overall, the loss curves do not indicate systematic training instability across folds. Some folds show mild overfitting tendencies or validation fluctuations, but these patterns are not consistent across folds. Because final model selection was based on validation macro F1-score with restored best weights, later fluctuations in validation loss were not necessarily reflected in the final evaluated model.

C Feature selection in the feature-based baseline

To complement the reported average number of selected features, we analyzed how often individual features were retained during feature selection across outer LOSO-CV folds. This analysis reflects feature-selection frequency rather than post hoc RF feature importance. We focus on the combined feature set, as this baseline achieved the highest feature-based performance and directly indicates which pupil diameter (PD)-statistics and fixation-characteristic features were retained when both feature groups were available to the model.

The most consistently selected features included both fixation characteristics and PD-statistics features (Fig. S2). Average fixation duration and the number of fixations were selected in all folds, whereas fixation durations were selected less frequently. Among the PD-statistics features, range, standard deviation, variance, samples until maximum, and the slope in the first half of the window were selected most consistently. These features primarily describe signal amplitude, variability, and early temporal change. Overall, the feature-selection pattern supports the interpretation that the combined baseline benefited from complementary information from both feature groups, while only a subset of the available PD statistics contributed consistently.

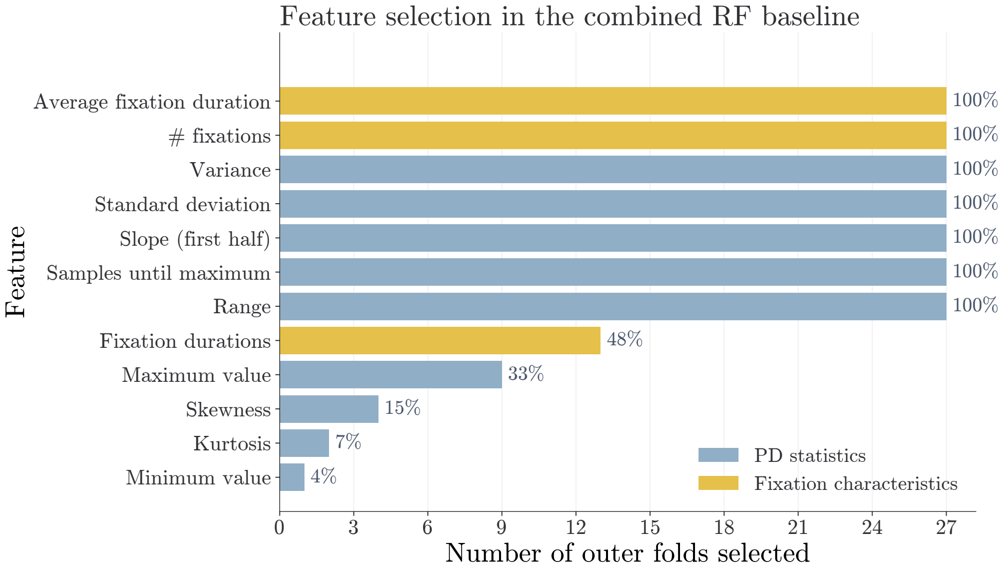

Figure S2. Feature-selection frequency for the combined random forest (RF) baseline using both pupil diameter (PD) statistics and fixation characteristics. Bars show how often each feature was selected across outer LOSO-CV folds. Percentages indicate the proportion of folds in which the feature was retained.

D Extended performance analysis of time-series-based models

D.1 Confidence intervals across outer folds

To complement the reported mean and standard deviation across outer LOSO-CV folds, we additionally computed 95% confidence intervals for selected performance metrics. Confidence intervals were calculated across outer folds, treating each held-out participant as one observation. For the VR goalkeeper dataset, this corresponds to $n = 27$ folds; for the ForDigitStress dataset, this corresponds to $n = 15$ folds. For each model and metric, the confidence interval was computed as

$$\bar{x} \pm t_{0.975,n-1} \cdot \frac{s}{\sqrt{n}},$$

where $\bar{x}$ denotes the mean performance across outer folds, s the corresponding standard deviation, n the number of folds, and $t_{0.975,n-1}$ the two-sided critical value of the t-distribution. The intervals provide descriptive uncertainty estimates for the mean fold-wise performance of each model configuration. Table S8 reports confidence intervals for the main feature-based baselines and representative time-series models discussed in the manuscript.

The confidence intervals support the descriptive results reported in the main manuscript. For the VR goalkeeper dataset, the representative time-series models show higher macro F1-scores than the feature-based baselines, with comparatively narrow confidence intervals. In contrast, the ForDigitStress models show substantially lower macro F1-scores, with confidence intervals remaining far below the best-performing VR configurations.

Table S8. Performance estimates with 95% confidence intervals (CIs) across outer leave-one-subject-out cross-validation (LOSO-CV) folds for the virtual reality (VR) goalkeeper dataset and the ForDigitStress dataset. Values are reported as percentages.

Dataset	Model	Metric	Mean ± SD	95% CI
VR goalkeeper	RF (PD statistics)	Macro F1	76.47 ± 10.99	$[72.13, 80.82]$
	RF (fixation characteristics)	Macro F1	78.46 ± 9.02	$[74.90, 82.03]$
	RF (combined)	Macro F1	83.64 ± 10.25	$[79.58, 87.69]$
	CNN (PD)	Macro F1	88.85 ± 8.86	$[85.35, 92.36]$
	ConvLSTM-3 (asymptotic model)	Macro F1	95.98 ± 5.83	$[93.67, 98.29]$
ForDigitStress	CNN (PD)	Macro F1	57.74 ± 6.96	$[53.89, 61.60]$
	LSTM-1 (PD)	Macro F1	53.07 ± 5.11	$[50.24, 55.90]$

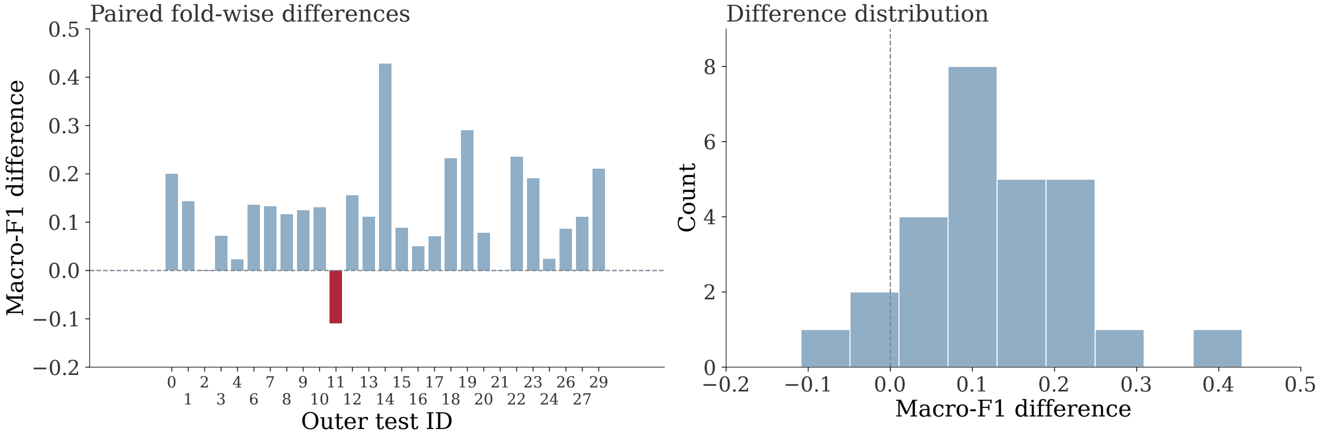

Figure S3. Distribution of paired fold-wise differences in macro F1-score between the best-performing deep learning model (ConvLSTM-3, asymptotic input) and the combined-feature random forest (RF) baseline. Each value corresponds to one held-out participant. The plot illustrates consistent performance improvements across folds and is not intended to assess distributional assumptions.

D.2 Detailed statistical comparison of model performance

To complement the descriptive performance evaluation, we conducted inferential statistical analysis on the VR goalkeeper dataset to assess whether observed performance differences between models are statistically meaningful. Statistical comparisons were performed on the outer folds of the nested LOSO-CV procedure, treating each held-out participant as an independent observation ($n = 27$). The evaluation metric was the macro-averaged F1-score. For each comparison, paired differences between models were computed across folds. Because the paired fold-wise differences may not follow a normal distribution and the number of held-out participants was limited, comparisons were conducted using the non-parametric Wilcoxon signed-rank test[3], which is more robust under small sample sizes and potentially non-normal paired differences. Two planned comparisons between representative deep learning (DL) models and corresponding feature-based baselines were evaluated. Because multiple statistical comparisons increase the risk of inflated Type I error rates, p-values were adjusted using the Holm correction method[4], which controls the family-wise error rate while being less conservative than the standard Bonferroni correction. Effect sizes are reported as rank-biserial correlations (r). First, the ConvLSTM-3 model using the asymptotic input signal was compared to the strongest RF baseline (combined feature set). The DL model achieved a higher mean macro-F1 score (95.98% ± 5.72%) than the baseline (83.64% ± 10.06%), corresponding to a mean paired difference of 12.3%. The Wilcoxon signed-rank test indicated a statistically significant difference ($W = 11$, $p < .001$, Holm-adjusted $p < .001$), with a large effect size ($r = -0.94$). Second, the CNN model using PD was compared to the RF baseline using PD-based features. The CNN achieved a higher mean macro-F1 score (88.85% ± 8.70%) than the baseline (76.47% ± 10.79%), corresponding to a mean paired difference of 12.4%. The Wilcoxon signed-rank test again indicated a statistically significant difference ($W = 27$, $p < .001$, Holm-adjusted $p < .001$), with a large effect size ($r = -0.86$). The distributions of paired fold-wise differences for both comparisons are shown in Figures S3 and S4, illustrating consistent performance improvements across participants. Overall, the results indicate that the evaluated DL models consistently outperformed the corresponding feature-based baselines across held-out participants, with large effect sizes observed for both comparisons.

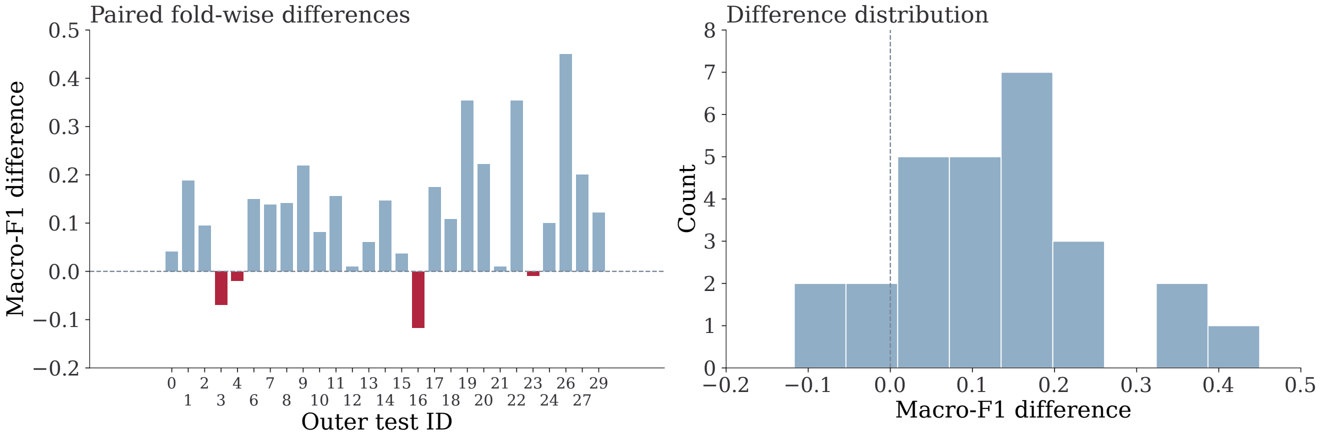

Figure S4. Distribution of paired fold-wise differences in macro F1-score between the pupil diameter (PD)-based convolutional neural network (CNN) model and the PD-based random forest (RF) baseline. Each value corresponds to one held-out participant. The plot illustrates consistent performance improvements across folds.

D.3 ROC and precision–recall analysis

To complement the reported F1-scores, we present receiver operating characteristic (ROC) and precision–recall (PR) curves for both datasets. For the VR goalkeeper dataset, Figure S5 shows the best-performing model for each input signal, enabling a comparison of signal-specific performance rather than architectural differences. Figure S6 presents the corresponding results for the ForDigitStress[5] dataset, where only PD-based models are available. Performance is summarized using the area under the ROC curve (AUC), which captures the trade-off between true positive and false positive rates across decision thresholds, and average precision, which summarizes the PR relationship. While AUC provides a general measure of class separability, average precision is particularly informative in imbalanced settings, as it emphasizes performance on the positive stress class[6,7]. Together, these plots provide an additional perspective on class separability and threshold-dependent model behavior across both datasets.

Consistent with the results presented in the main manuscript, the curves indicate strong separability across input signals for the VR goalkeeper dataset, with ROC curves close to the upper-left region and PR curves maintaining high precision over a broad recall range. Differences between models and signals are small in absolute terms and mainly occur in specific operating regions, with the strongest ConvLSTM-3 models showing slight advantages at high recall.

For the ForDigitStress dataset, the curves indicate lower separability and stronger threshold dependence. ROC curves intersect across architectures, showing that model ranking depends on the chosen operating point: the CNN performs comparatively better at low false positive rates, whereas recurrent architectures can become more favorable at more permissive thresholds. The PR curves further show that maintaining high precision becomes difficult as recall increases, consistent with the class imbalance and reduced stress-class recall observed in the confusion matrix.

D.4 Computational complexity analysis

To assess the computational cost of the evaluated models, we conducted a post hoc complexity analysis based on the best-performing configurations identified during hyperparameter optimization. For each outer fold of the LOSO-CV procedure, the corresponding model architecture was reconstructed using the best Optuna trial for the respective held-out participant. The reconstruction followed the same preprocessing and input transformation pipeline as used during training, ensuring consistency between training and evaluation settings. No retraining was performed. For each reconstructed model, we measured the number of trainable and non-trainable parameters, estimated memory requirements, and inference latency based on forward-pass evaluation. The reported values correspond to summary statistics aggregated across outer folds. All models were originally trained with a single NVIDIA RTX 3080 GPU (10 GB VRAM) and Intel Xeon CPUs. Computational complexity evaluation was conducted separately on a local machine (Apple M3 Pro chip, 36 GB RAM). Table S9 summarizes the computational complexity of representative model configurations corresponding to the main experimental results.

The results in Table S9 show clear differences in computational cost between the evaluated model configurations. The CNN-based model exhibits a low parameter count (0.25 ± 0.21 M) and memory requirement (0.94 ± 0.79 MB), together with the lowest inference time (4.70 ± 0.73 ms per sample). The LSTM-1 model has a lower parameter count (0.14 ± 0.27 M) and memory usage (0.54 ± 1.03 MB), but substantially higher inference latency (345.88 ± 36.62 ms per sample). ConvLSTM-based models show higher parameter counts and memory requirements, with the ConvLSTM-1 model reaching 0.99 ± 1.32 M parameters and ConvLSTM-3 reaching 3.60 ± 4.03 M parameters, and corresponding memory usage of 3.76 ± 5.04 MB and 13.74 ± 15.37 MB, respectively. Inference latency increases accordingly, from 74.17 ± 25.99 ms per sample for

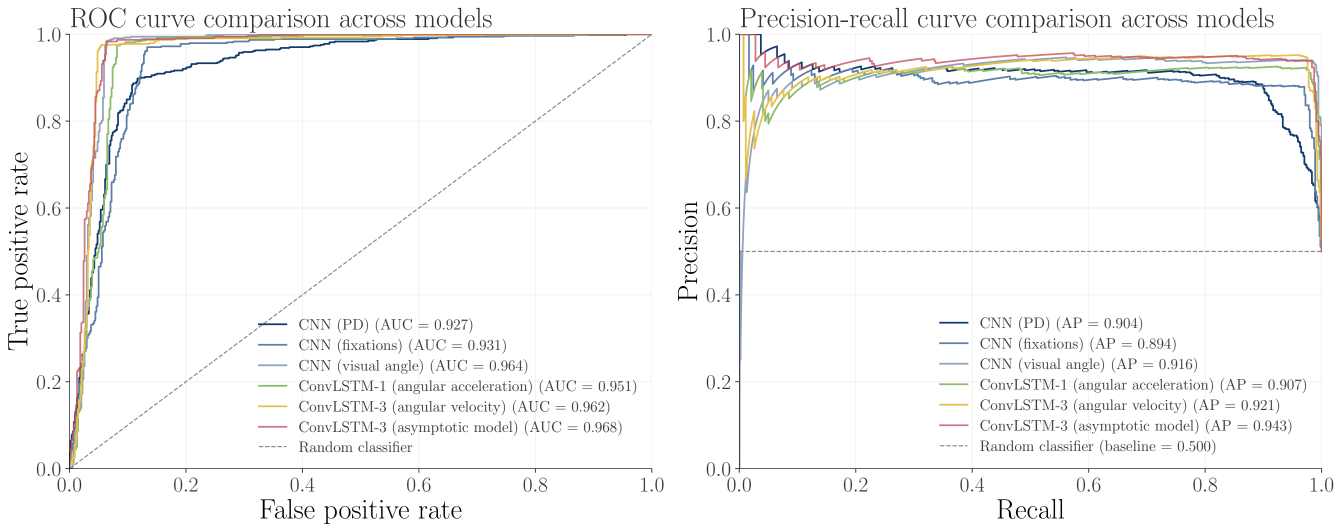

Figure S5. Receiver operating characteristic (ROC; left) and precision–recall (PR; right) curves for the virtual reality (VR) goalkeeper dataset showing the best-performing model for each input signal. The area under the ROC curve (AUC) quantifies separability across decision thresholds, reflecting the trade-off between true positive rate and false positive rate. Average precision (AP) summarizes the precision–recall trade-off and emphasizes performance on the positive stress class. The dashed diagonal line in the ROC plot indicates the performance of a random classifier; the dashed horizontal line in the PR plot indicates baseline precision.

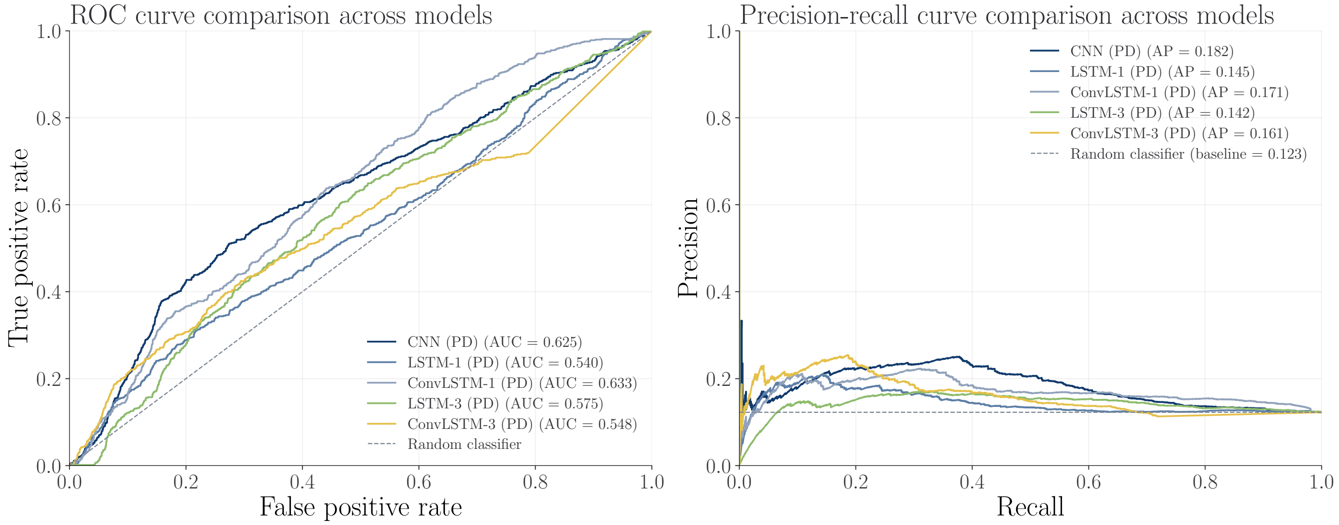

Figure S6. Receiver operating characteristic (ROC; left) and precision–recall (PR; right) curves for the ForDigitStress dataset showing all pupil diameter (PD)-based models. The area under the ROC curve (AUC) quantifies separability across decision thresholds, reflecting the trade-off between true positive rate and false positive rate. Average precision (AP) summarizes the precision–recall trade-off and emphasizes performance on the positive stress class. The dashed diagonal line in the ROC plot indicates the performance of a random classifier; the dashed horizontal line in the PR plot indicates baseline precision.

Table S9. Computational complexity of the evaluated architectures on the virtual reality (VR) goalkeeper dataset (asymptotic model input). Values are reported as mean ± standard deviation across leave-one-subject-out cross-validation (LOSO-CV) folds.

Model	Params (M)	Memory (MB)	Weights (MB)	Latency (ms)
CNN	0.25 ± 0.21	0.94 ± 0.79	2.87 ± 2.36	4.70 ± 0.73
LSTM-1	0.14 ± 0.27	0.54 ± 1.03	1.64 ± 3.10	345.88 ± 36.62
ConvLSTM-1	0.99 ± 1.32	3.76 ± 5.04	11.37 ± 15.09	74.17 ± 25.99
LSTM-3	1.94 ± 1.18	7.38 ± 4.52	23.08 ± 15.24	1056.51 ± 72.02
ConvLSTM-3	3.60 ± 4.03	13.74 ± 15.37	41.85 ± 46.00	217.53 ± 64.49

ConvLSTM-1 to 217.53 ± 64.49 ms per sample for ConvLSTM-3. The LSTM-3 model has 1.94 ± 1.18 M parameters and a memory requirement of 7.38 ± 4.52 MB, with the highest inference latency among all evaluated models (1056.51 ± 72.02 ms

per sample).

E Additional temporal attribution analyses

This section provides additional attribution analyses complementing the temporal occlusion results presented in the main manuscript. To improve interpretability and better understand which parts of the input signals contributed to model predictions, we performed temporal attribution analysis on trained outer-fold models using held-out test samples. No model retraining was performed for this analysis.

We used two complementary attribution approaches: vanilla gradient and temporal occlusion sensitivity. For the gradient-based analysis, we computed vanilla saliency maps by taking the absolute value of the gradient of the model output score for the predicted class with respect to the input signal saliency[8]. Thus, larger values indicate time points at which small changes in the input would be expected to have a stronger local effect on the model's prediction. As a perturbation-based comparison, we applied temporal occlusion sensitivity by systematically masking local temporal windows and measuring the resulting decrease in the predicted class score[9]. Occlusion was performed with a window size of 15 samples, a stride of 5 samples, and mean-value replacement within the masked window.

For both methods, attribution maps were reduced to one-dimensional temporal relevance profiles and aggregated across held-out samples. In addition to the comparative occlusion analysis presented in the main manuscript for correctly classified stress samples using the PD-based CNN models, the supplementary analyses provide class-specific attribution profiles, saliency-based analyses, individual attribution examples, and additional attribution results for the asymptotic-model-based ConvLSTM-3. To enable a structured comparison, we analyzed three representative models: (i) the best-performing model on the VR goalkeeper dataset, namely ConvLSTM-3 with the asymptotic model input signal, (ii) a PD-based CNN on the same dataset, and (iii) a PD-based CNN on the ForDigitStress dataset.

Figures S7 and S8 provide additional attribution results for the best-performing VR model (ConvLSTM-3 with asymptotic model input signal). Consistent with the comparative attribution analysis presented in the main manuscript, both saliency and occlusion analysis indicate that the most relevant temporal regions are concentrated in the early part of the analyzed segment. Because the model input is represented as 25 temporal segments with approximately 18 samples each, the projected attribution profiles exhibit a repeated geometric pattern. This structure reflects the segmented asymptotic-model representation rather than intrinsic periodicity of the physiological signal.

For the asymptotic model, which captures the temporal evolution and stabilization of gaze behavior, the attribution patterns are compatible with the model relying on structured gaze dynamics during the preparation phase shortly after completion of the cognitive task. Compared with the PD-based CNN, the attribution profiles are more sharply structured and temporally localized, consistent with the superior classification performance of the asymptotic-model-based ConvLSTM-3. While aggregated attribution profiles emphasize early temporal regions, individual samples (Figure S9) may still exhibit informative contributions across the entire segment.

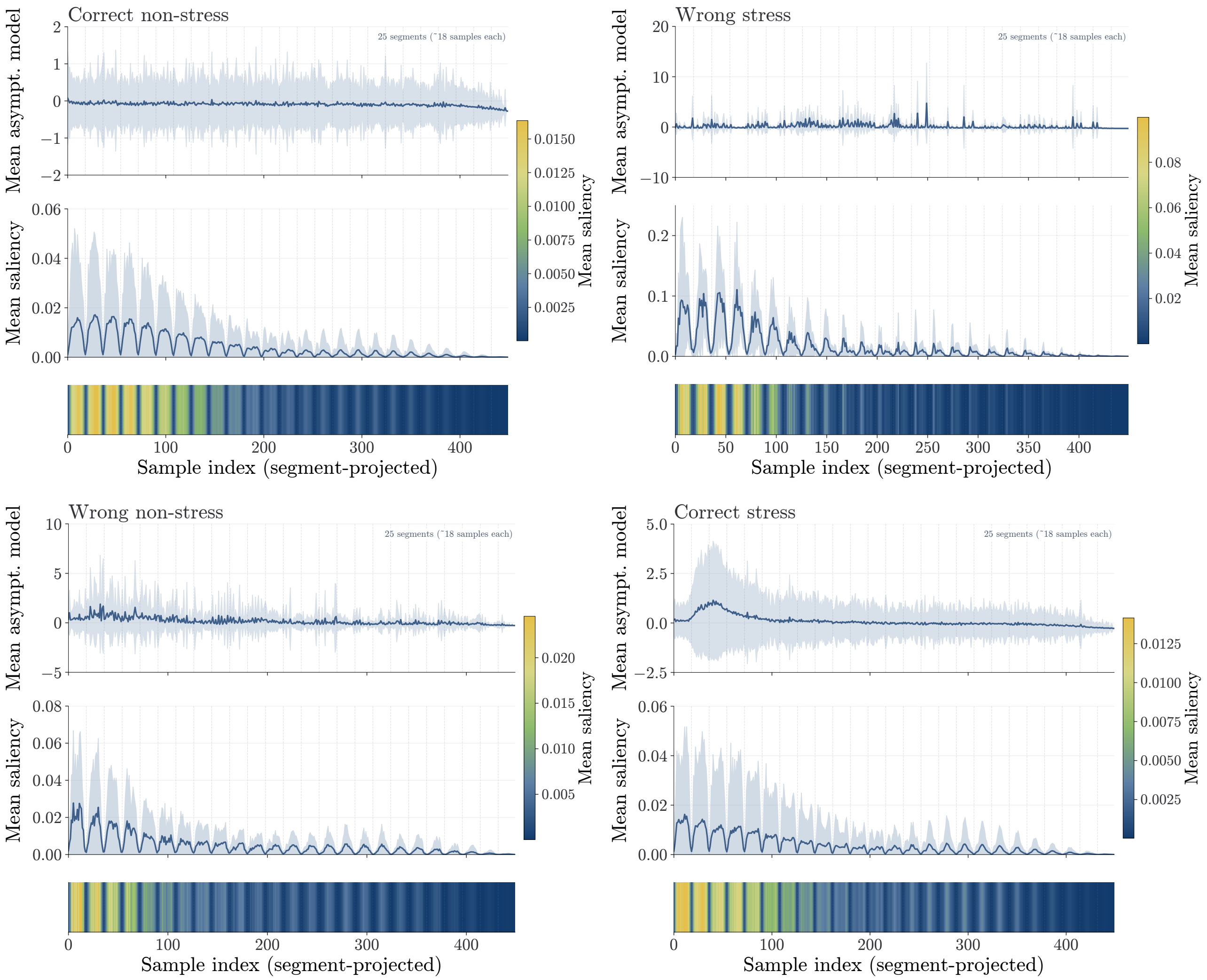

Figure S7. Mean saliency profiles for the convolutional long short-term memory (ConvLSTM)-3 using the asymptotic model (asympt. model) input on the virtual reality (VR) goalkeeper dataset. Panels correspond to correct non-stress (top-left), wrong stress (top-right), wrong non-stress (bottom-left), and correct stress (bottom-right). The input is represented as 25 temporal segments (approximately 18 samples each), resulting in a repeated geometric structure after projection to temporal order. This pattern reflects the segmented asymptotic model representation rather than physiological periodicity. Across all cases, saliency is highest in the early part of the segment, with more distinct and structured peaks for correct predictions.

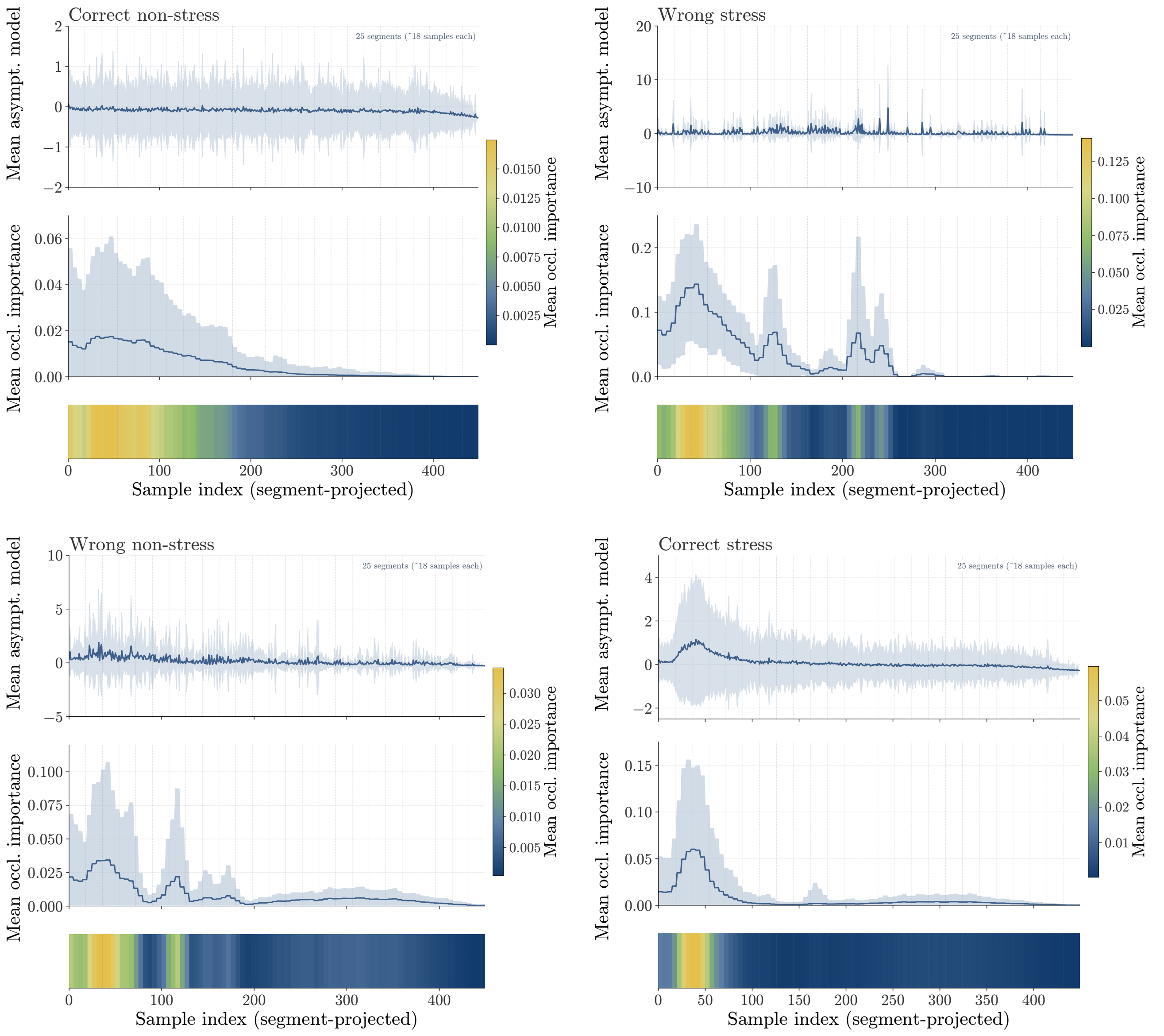

Figure S8. Mean occlusion-importance (mean occl. importance) profiles for the convolutional long short-term memory (ConvLSTM)-3 using the asymptotic model (asympt. model) input on the virtual reality (VR) goalkeeper dataset. Panels correspond to correct non-stress (top-left), wrong stress (top-right), wrong non-stress (bottom-left), and correct stress (bottom-right). In each panel, the upper time series shows the mean input signal across samples, and the shaded band indicates ± one standard deviation across samples. The middle time series shows the mean occlusion-importance profile, with the shaded band indicating ± one standard deviation across samples; the lower bound is clipped at zero because occlusion importance is shown on a non-negative scale. The color-coded representation below shows the same mean occlusion-importance values over time. Masking early temporal regions leads to the strongest decrease in predicted class score, consistent with the comparative attribution analysis presented in the main manuscript.

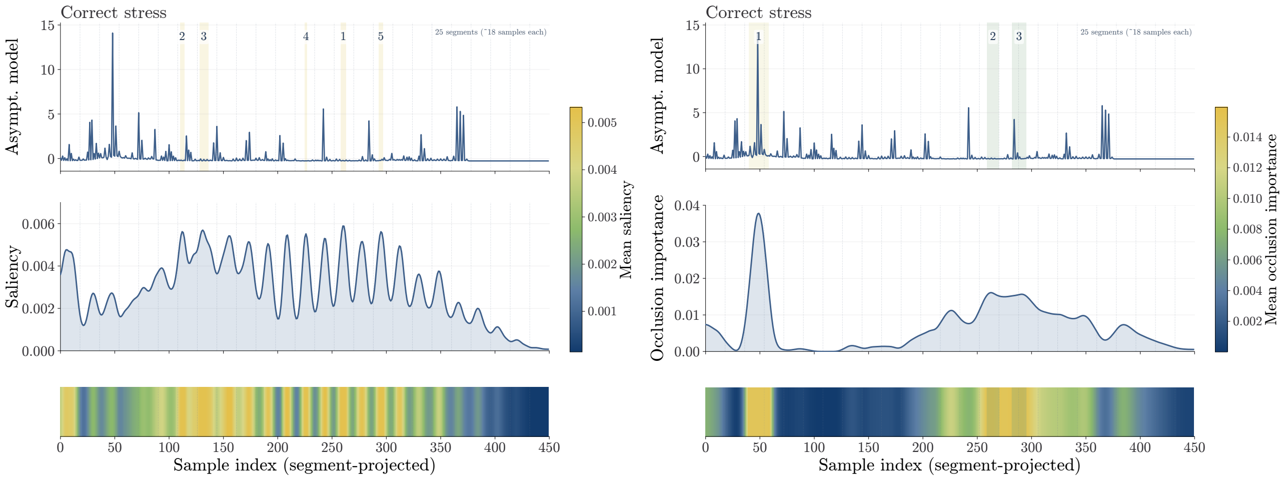

Figure S9. Representative individual attribution example for the asymptotic-model-based convolutional long short-term memory (ConvLSTM)-3 on the virtual reality (VR) goalkeeper dataset (correct stress trial, subject 26, sample 31). The left panel shows saliency and the right panel occlusion-based importance for the same sample. Highlighted regions indicate up to five contiguous temporal windows with the highest smoothed attribution values, defined from the upper 10% of normalized attribution values and ranked by their mean attribution. For saliency, these regions mark signal portions where small input changes are expected to have the strongest local effect on the predicted class score; for occlusion, they mark signal portions whose masking most strongly reduced the predicted class score. In addition to early relevance, both methods highlight informative regions in the middle and later parts of the segment, illustrating that later temporal information can contribute to correct predictions in individual trials.

To assess whether the primary temporal relevance pattern is specific to the asymptotic model, we additionally analyzed the PD-based CNN on the VR goalkeeper dataset. The corresponding attribution results (Figures S10 and S11) show a similar temporal emphasis, with higher relevance in the early part of the segment, consistent with the comparative attribution analysis presented in the main manuscript. Compared with the asymptotic-model-based ConvLSTM-3, the attribution profiles are less sharply structured and temporally localized, consistent with the lower overall performance of the PD-based CNN. The early attribution peak is compatible with a task-evoked pupillary response extending into the analyzed segment because of its close temporal proximity to the preceding cognitive task. Taken together, the attribution results from both the asymptotic-model and PD-based analyses suggest that stress-related information is reflected in gaze-derived temporal dynamics as well as pupillary responses, although the exact physiological mechanisms cannot be confirmed based on the present analysis.

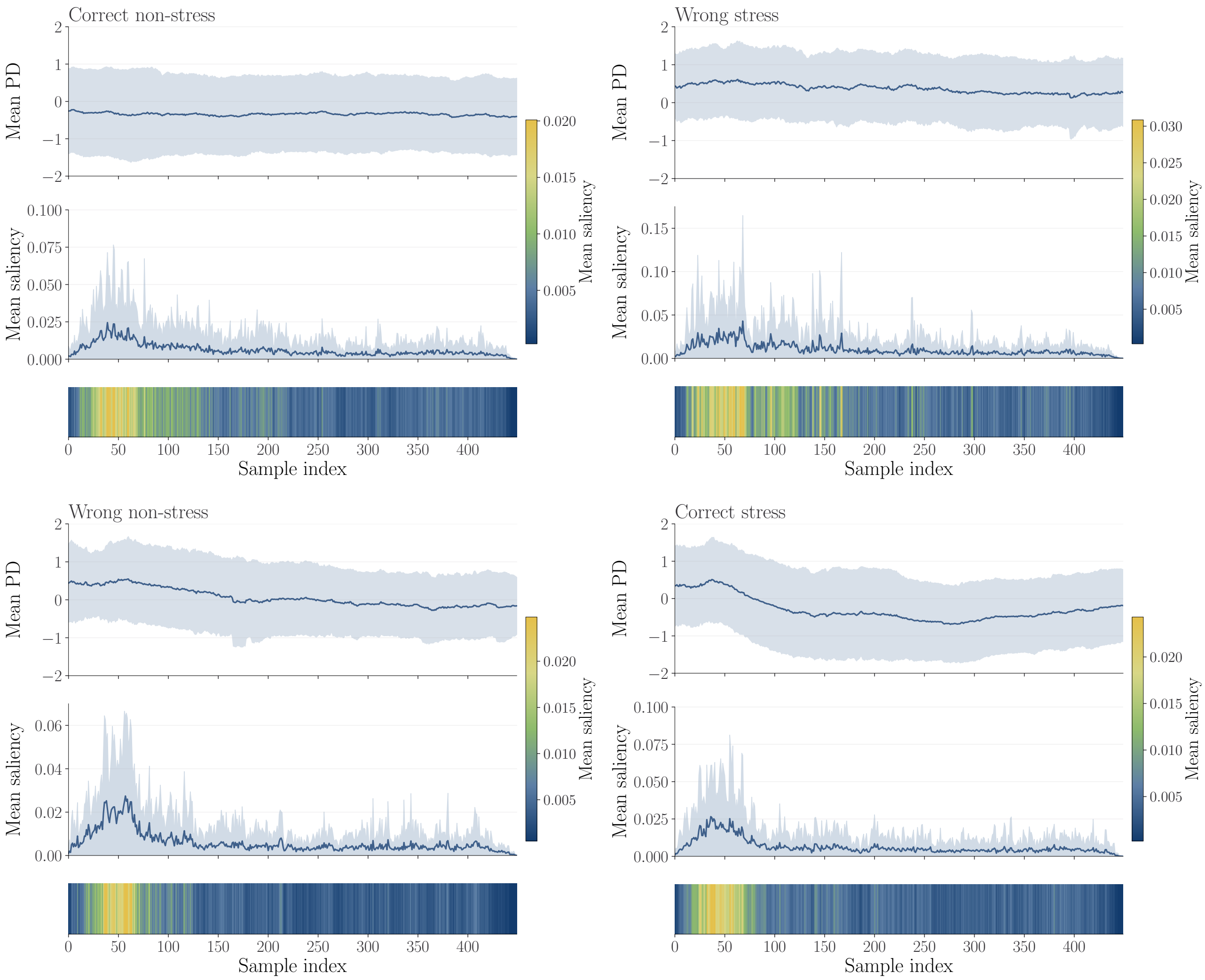

Figure S10. Mean saliency profiles for the convolutional neural network (CNN) model with pupil diameter (PD) input on the virtual reality (VR) goalkeeper dataset. Panels correspond to correct non-stress (top-left), wrong stress (top-right), wrong non-stress (bottom-left), and correct stress (bottom-right). In each panel, the upper time series shows the mean input signal across samples, and the shaded band indicates ± one standard deviation across samples. The middle time series shows the mean saliency profile, with the shaded band indicating ± one standard deviation across samples; the lower bound is clipped at zero because saliency is shown on a non-negative scale. The color-coded representation below shows the same mean saliency values over time. Across all cases, saliency is highest in the early part of the segment, although the profiles are less sharply structured than for the convolutional long short-term memory (ConvLSTM)-3 (asymptotic model). Correct predictions show somewhat clearer early peaks, indicating that the CNN also relies primarily on early post-task pupillary information, but with less distinct temporal structure.

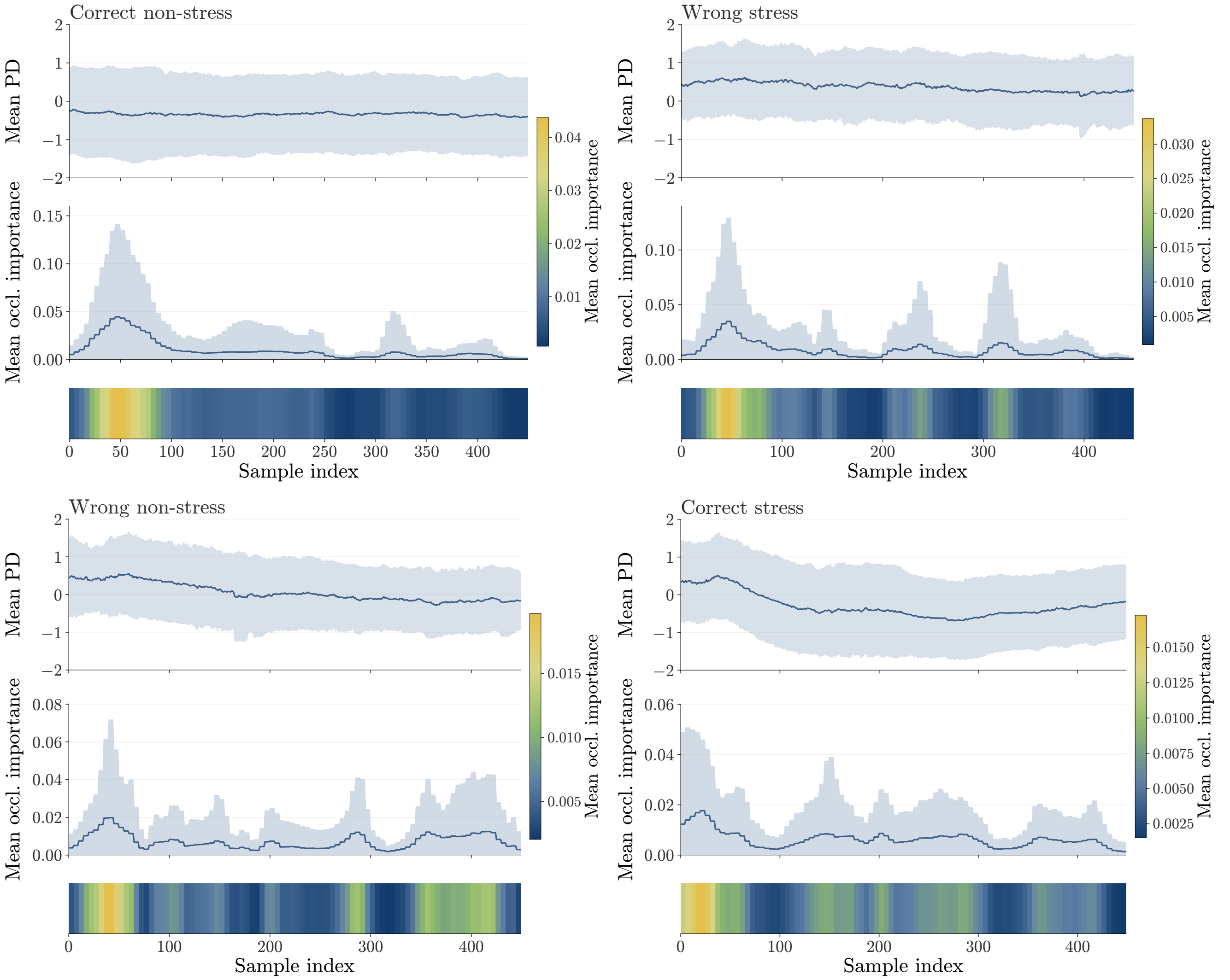

Figure S11. Mean occlusion-importance (mean occl. importance) profiles for the convolutional neural network (CNN) model with pupil diameter (PD) input on the virtual reality (VR) goalkeeper dataset. Panels correspond to correct non-stress (top-left), wrong stress (top-right), wrong non-stress (bottom-left), and correct stress (bottom-right). In each panel, the upper time series shows the mean input signal across samples, and the shaded band indicates $\pm$ one standard deviation across samples. The middle time series shows the mean occlusion-importance profile, with the shaded band indicating $\pm$ one standard deviation across samples; the lower bound is clipped at zero because occlusion importance is shown on a non-negative scale. The color-coded representation below shows the same mean occlusion-importance values over time. Masking early temporal regions leads to the strongest decrease in predicted class score across all cases, indicating that the model relies primarily on information from the beginning of the segment. Compared with the convolutional long short-term memory (ConvLSTM)-based asymptotic model, the profiles are less sharply structured, but they support the same general emphasis on early post-task pupillary information.

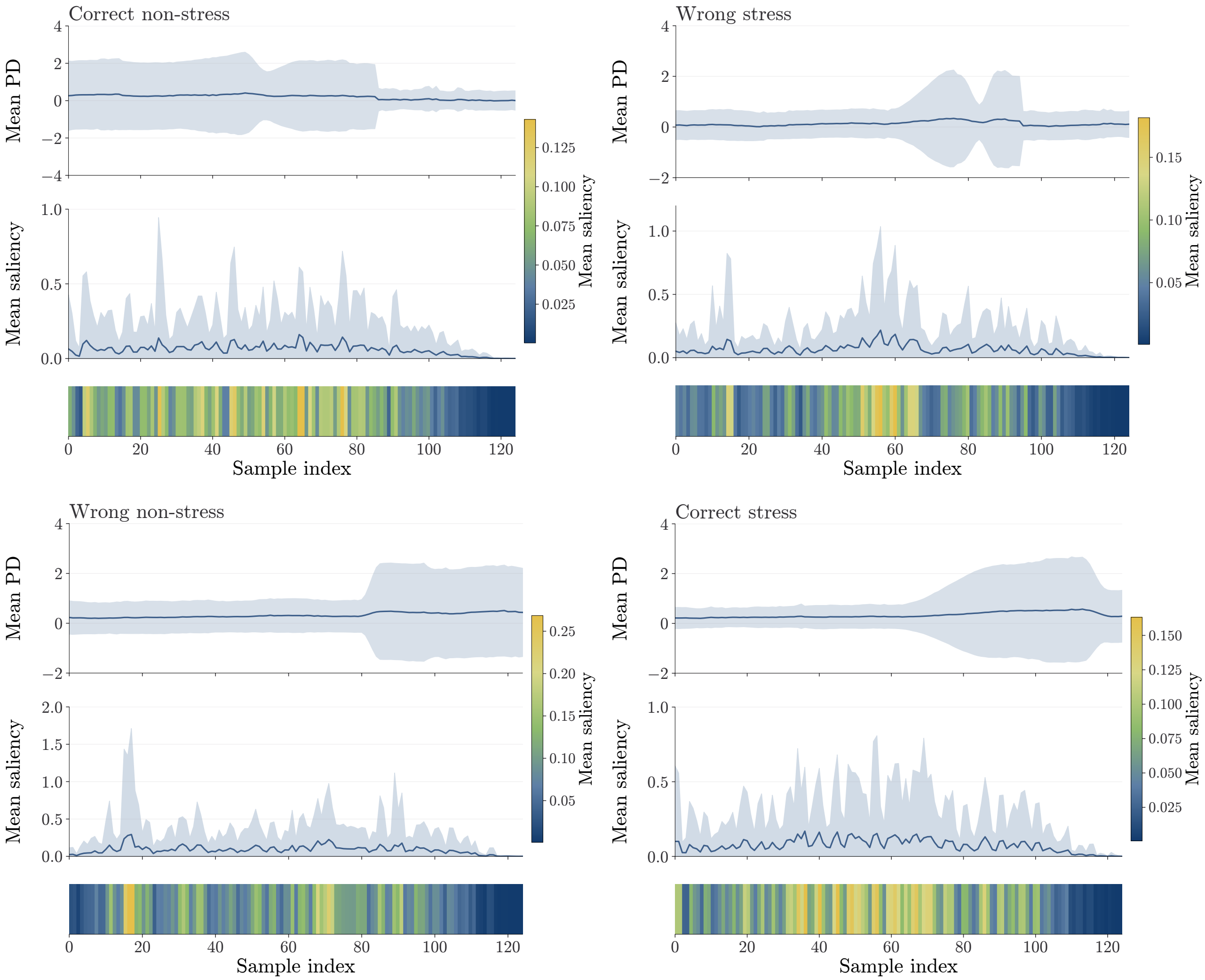

Figure S12. Mean saliency profiles for the convolutional neural network (CNN) model with pupil diameter (PD) input on the ForDigitStress dataset. Panels correspond to correct non-stress (top-left), wrong stress (top-right), wrong non-stress (bottom-left), and correct stress (bottom-right). In each panel, the upper time series shows the mean input signal across samples, and the shaded band indicates ± one standard deviation across samples. The middle time series shows the mean saliency profile, with the shaded band indicating ± one standard deviation across samples; the lower bound is clipped at zero because saliency is shown on a non-negative scale. The color-coded representation below shows the same mean saliency values over time. Saliency is concentrated early in the segment but is more diffuse than in the VR dataset, consistent with lower and less stable performance.

Figures S12 and S13 provide additional attribution results for the PD-based CNN on the ForDigitStress dataset. Consistent with the comparative attribution analysis presented in the main manuscript, attribution patterns are more broadly distributed across the segment than in the VR goalkeeper dataset, with relevance spanning early-to-middle and middle temporal regions rather than a single dominant onset. A consistent pattern across both saliency and occlusion analysis is reduced attribution toward the end of the window, indicating that discriminative information is represented more strongly in earlier portions of the analyzed segments.

Because windows are extracted with overlap from continuous recordings, similar signal content may occur at different temporal positions across windows. The observed attribution patterns therefore reflect the distribution of informative features within individual windows rather than an intrinsic lack of relevance of later signal components. Overall, the attribution profiles remain more diffuse and variable across samples, which is plausible given the less well-defined and more continuous stressor in the interview setting, where responses to individual questions are likely subtler and less temporally localized than the more clearly evoked responses to the cognitive task in the VR goalkeeper dataset.

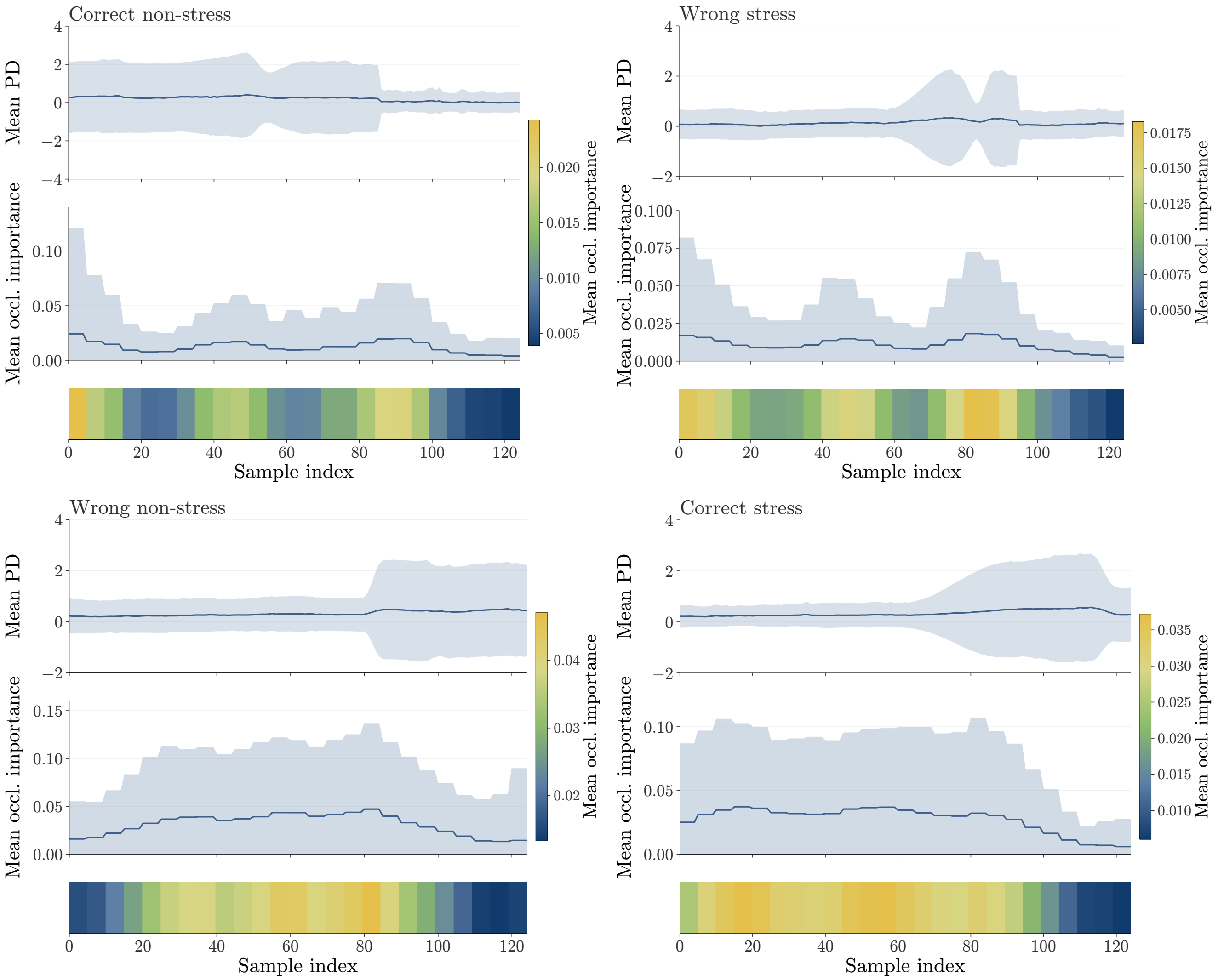

Figure S13. Mean occlusion-importance (mean occl. importance) profiles for the convolutional neural network (CNN) model with pupil diameter (PD) input on the ForDigitStress dataset. In each panel, the upper time series shows the mean input signal across samples, and the shaded band indicates ± one standard deviation across samples. The middle time series shows the mean occlusion-importance profile, with the shaded band indicating ± one standard deviation across samples; the lower bound is clipped at zero because occlusion importance is shown on a non-negative scale. The color-coded representation below shows the same mean occlusion-importance values over time. Masking early temporal regions leads to the strongest reduction in predictive information, confirming that early signal components are most relevant for classification.

F Error analysis

F.1 Subject-level error analysis

To further investigate model errors beyond aggregate performance metrics, we conducted a subject-level error analysis for both datasets, focusing on whether participant-specific difficulty is consistent across models and input signals. Here, subject "difficulty" refers to lower macro F1-scores for a given participant. We first analyzed agreement in subject difficulty by computing Spearman correlations of subject-level macro F1-scores across model configurations. Higher correlations indicate stronger agreement between configurations in the relative difficulty of individual participants.

Because the full VR goalkeeper model–signal matrix combines architecture and input-signal effects, Figure S14 shows two reduced representations for this dataset. The left panel shows one representative configuration per architecture, selected using the best-performing input signal for that architecture. This view emphasizes architecture-level agreement. The middle panel shows one representative configuration per input signal, selected using the best-performing architecture for that signal. This view emphasizes input-signal-level agreement. The right panel shows all evaluated PD-based architectures for the ForDigitStress dataset, where the input signal is fixed.

In the architecture-level VR goalkeeper representation (Fig. S14, left), agreement is strongest among the CNN and

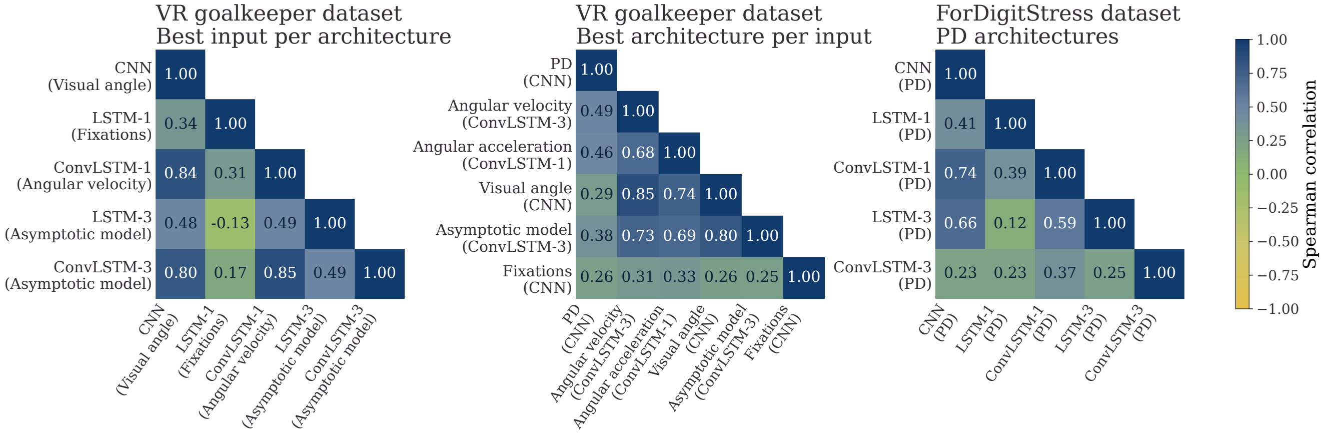

Figure S14. Spearman correlation of subject-level macro F1-scores across representative model configurations. Left: VR goalkeeper dataset with one configuration per architecture, selected using the best-performing input signal for each architecture. Middle: VR goalkeeper dataset with one configuration per input signal, selected using the best-performing architecture for each signal. Right: ForDigitStress dataset across all evaluated PD-based architectures. Only the lower triangle is shown because the correlation matrices are symmetric. Higher values indicate stronger agreement in relative subject difficulty across configurations.

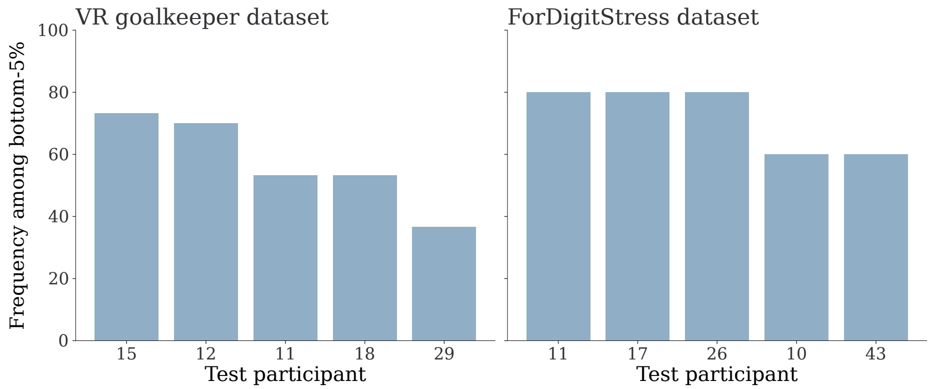

Figure S15. Frequency of participants appearing among the five lowest-performing subjects across model configurations. Left: VR goalkeeper dataset across all model and input-signal combinations. Right: ForDigitStress dataset across all evaluated PD-based models. Frequencies are expressed relative to the number of analyzed configurations in each dataset.

ConvLSTM-based configurations, indicating that these models tend to identify similar participants as relatively easier or harder to classify. In contrast, agreement with the LSTM-based configurations is lower, suggesting that subject difficulty is not entirely architecture-independent.

In the input-signal-level VR goalkeeper representation (Fig. S14, middle), dynamic gaze-derived signals show comparatively high agreement with each other, particularly visual angle, angular velocity, angular acceleration, and the asymptotic-model representation. In contrast, PD and fixation-based configurations show lower or less consistent agreement with several other input signals. This suggests that participant-specific difficulty is more consistently expressed for the stronger dynamic gaze representations than for more static input signals.

For the ForDigitStress dataset (Fig. S14, right), agreement in subject difficulty is less consistently structured across architectures. Some architecture pairs show moderate-to-high agreement, such as CNN and ConvLSTM-1, whereas other pairs show weak correlations. This indicates that participant-specific difficulty is also present in this dataset, but that the relative ranking of difficult participants depends more strongly on the model architecture.

To complement the rank-correlation analysis, Figure S15 shows how often participants appear among the five lowest-performing cases across model configurations.

For the VR goalkeeper dataset, a subset of participants appears repeatedly among the five lowest-performing subjects across many model–signal combinations, indicating stable subject-level difficulty. The strongest cases occur in 70–73% of the analyzed combinations. For the ForDigitStress dataset, the same pattern is also visible despite the smaller number of evaluated model configurations: several participants appear among the five lowest-performing subjects in 80% of the models, and additional participants appear in 60%. Thus, difficult participants are present in both datasets, and the relative recurrence of the most

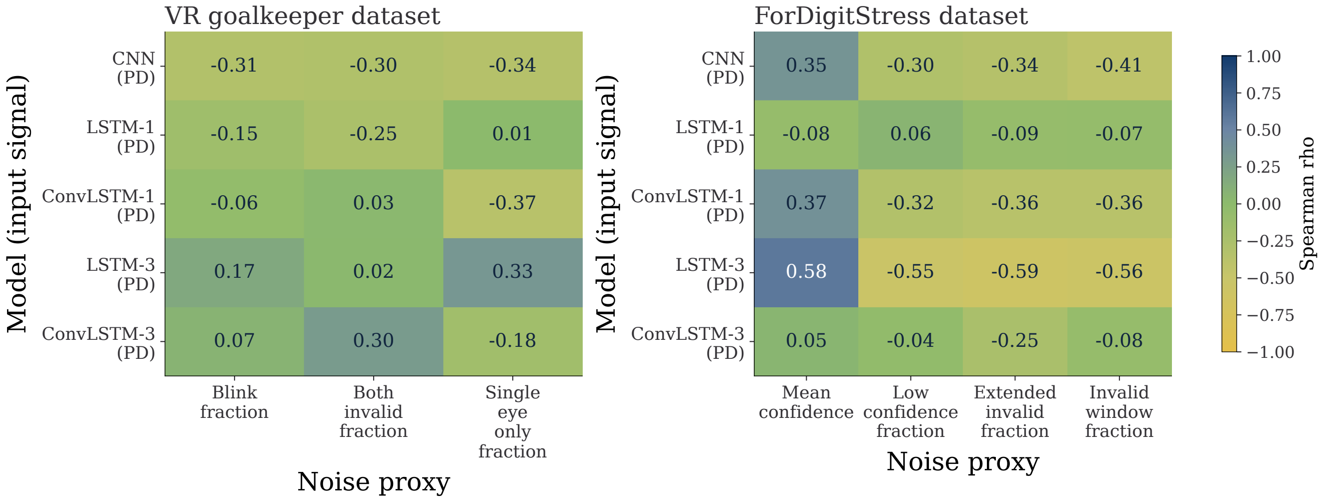

Figure S16. Spearman correlation between subject-level macro F1-scores and noise-related metrics for pupil diameter (PD)-based models. Left: virtual reality (VR) goalkeeper dataset. Noise proxies include blink fraction, both-invalid fraction (proportion of samples where both eyes are invalid), and single-eye-only fraction (proportion of samples with valid data from only one eye). Right: ForDigitStress dataset. Noise proxies include mean confidence, low-confidence fraction, extended-invalid fraction (proportion of samples marked invalid after confidence-based padding), and invalid-window fraction (proportion of candidate windows rejected during extraction). Negative correlations for confidence-like metrics and positive correlations for invalidity-related metrics would indicate lower performance with poorer signal quality.

difficult ForDigitStress participants is at least as pronounced as in the VR goalkeeper dataset. However, this comparison should be interpreted with caution because the ForDigitStress analysis includes only five PD-based model configurations, whereas the VR analysis spans 30 model–signal combinations.

F.2 Noise-related error analysis

To further investigate the influence of signal quality on model performance, we analyzed the relationship between PD noise and subject-level classification performance for both datasets. Because the datasets differ in recording setup and preprocessing, dataset-specific noise proxies were used. For the VR goalkeeper dataset, noise was quantified using preprocessing-derived metrics such as blink fraction and fractions of invalid samples (e.g., both eyes invalid, single-eye-only tracking), reflecting tracking interruptions and signal instability. For the ForDigitStress dataset, noise proxies were derived from the eye-tracker confidence signal and the PD window-extraction procedure. Specifically, we quantified mean confidence, the fraction of low-confidence samples (low-confidence fraction), the fraction of samples rendered invalid after confidence-based padding (extended-invalid fraction), and the fraction of candidate windows rejected during extraction (invalid-window fraction). These metrics capture both sample-level signal quality and window-level usability for modeling. Figure S16 shows the Spearman correlation between subject-level macro F1-scores and noise-related metrics for both datasets.

For the VR goalkeeper dataset (Fig. S16, left), correlations are generally weak and inconsistent across models, indicating that the analyzed PD noise proxies explain little of the subject-level performance variability. Some configurations show a slight tendency toward lower performance at higher noise levels, particularly the PD-based CNN and ConvLSTM-1 models, but this pattern is not consistent across architectures.

For the ForDigitStress dataset (Fig. S16, right), associations between signal-quality proxies and performance are more visible for specific architectures, but they remain model-dependent. In particular, the LSTM-3 model shows the clearest pattern, with higher mean confidence associated with higher macro F1-scores and higher fractions of low-quality or invalid samples associated with lower macro F1-scores. The CNN and ConvLSTM-1 models show similar but more moderate tendencies for some metrics, whereas the ConvLSTM-3 model exhibits comparatively weak associations.

Overall, the analyses suggest that signal quality contributes to performance variability, particularly in the ForDigitStress dataset, but does not fully account for the observed dataset-level performance differences. The model-dependent correlation patterns suggest that architectures differ in their sensitivity to signal quality, rather than signal quality exerting a uniform effect on classification performance.

F.3 VR goalkeeper dataset: sample-order error analysis

To assess whether classification performance of the ConvLSTM-3 (asymptotic model) for the VR goalkeeper dataset varies systematically over time, we analyzed the classification error rate as a function of the original sample order within each subject.

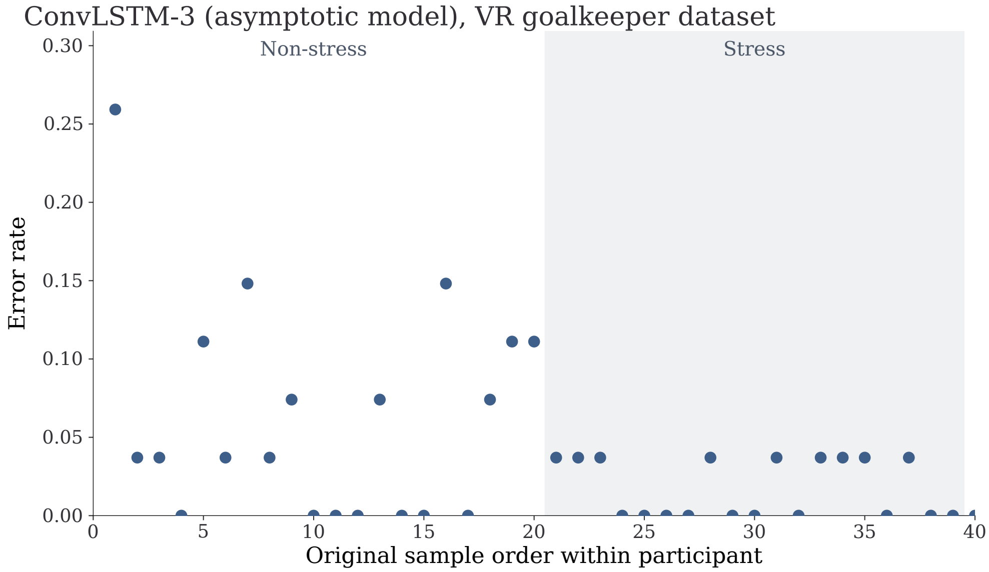

Figure S17. Classification error rate of the convolutional long short-term memory (ConvLSTM)-3 (asymptotic model) as a function of sample order within each subject for the virtual reality (VR) goalkeeper dataset. The shaded region indicates the stress phase.

Fig. S17 shows the classification error rate for each shot position, aggregated across participants. The shaded region indicates the stress phase. The results show no increase in error rates over time. Instead, the highest error rate occurs at the very first shot, with a value substantially above all subsequent positions. For later shots, error rates remain low overall and do not exhibit a systematic upward trend. A comparison between phases shows that error rates are generally lower during the stress phase than during the non-stress phase. This pattern is consistent with the aggregated confusion matrix, which shows substantially fewer misclassifications for stress than for non-stress samples. The elevated error rate at the first shot may indicate a possible initial adaptation or transition effect when participants enter the final task environment. Beyond this initial point, the absence of increasing error rates across sample order indicates that fatigue-related effects are not evident in the data and are unlikely to account for the observed error patterns.

G Additional implementation details for robustness experiments

This section provides additional implementation details for the robustness experiments described in the main manuscript.

Because the ForDigitStress dataset does not provide calibrated PD measurements in physical units, scale-dependent preprocessing steps used in the VR goalkeeper preprocessing pipeline (e.g., dilation-speed filtering and out-of-bounds removal) could not be applied consistently. These preprocessing steps rely on physiologically interpretable pupil measurements and would otherwise require arbitrary thresholds. To assess whether such technical differences contribute substantially to the observed performance gap between datasets, we performed additional robustness experiments on the VR goalkeeper dataset by progressively approximating selected recording and preprocessing characteristics of the ForDigitStress recordings while keeping the underlying dataset and task unchanged. The robustness experiments therefore approximate selected technical differences between datasets but do not reproduce differences in task design, annotation quality, class imbalance, participant characteristics, or hardware-specific noise.

The preprocessing pipeline was progressively simplified by (i) reducing the sampling rate to 2.5×10^1 Hz, (ii) using monocular right-eye pupil signals instead of binocular averaging, and (iii) removing scale-dependent filtering steps. Missing or invalid PD samples were first interpolated and resampled to the regular 9.0×10^1 Hz time axis using piecewise cubic Hermite interpolation, followed by polyphase downsampling to 2.5×10^1 Hz using `scipy.signal.resample_poly`. Consequently, five-second windows in the downsampled conditions contained 125 samples instead of 450 samples.

PD baseline correction was recomputed independently for each robustness condition using the corresponding signal representation and sampling rate, and the baseline segment was always processed identically to the associated signal condition.

Because the robustness analysis focused on the best-performing PD-based classification pipeline, only the CNN architecture was evaluated. The same participant subset as in the main VR goalkeeper analysis was used. Apart from the preprocessing modifications described above, the training and evaluation pipeline remained unchanged and followed the same nested LOSO-CV procedure and hyperparameter optimization strategy described in the main manuscript.

References

1. Akiba, T., Sano, S., Yanase, T., Ohta, T. & Koyama, M. Optuna: A next-generation hyperparameter optimization framework. In *Proceedings of the 25th ACM SIGKDD International Conference on Knowledge Discovery & Data Mining*, KDD '19, 2623–2631, DOI: 10.1145/3292500.3330701 (Association for Computing Machinery, New York, NY, USA, 2019).
2. Lin, T.-Y., Goyal, P., Girshick, R., He, K. & Dollár, P. Focal loss for dense object detection. In *Proceedings of the IEEE International Conference on Computer Vision*, 2980–2988, DOI: 10.1109/TPAMI.2018.2858826 (2017).
3. Wilcoxon, F. Individual comparisons by ranking methods. *Biom. Bull.* **1**, 80–83, DOI: 10.2307/3001968 (1945).
4. Holm, S. A simple sequentially rejective multiple test procedure. *Scand. J. Stat.* **6**, 65–70, DOI: 10.2307/4615733 (1979).
5. Heimerl, A. *et al.* The ForDigitStress dataset: A multi-modal dataset for automatic stress recognition. *IEEE Trans. Affect. Comput.* **16**, 1219–1234, DOI: 10.1109/TAFFC.2024.3501400 (2025).
6. Fawcett, T. An introduction to roc analysis. *Pattern Recognit. Lett.* **27**, 861–874, DOI: 10.1016/j.patrec.2005.10.010 (2006).
7. Saito, T. & Rehmsmeier, M. The precision-recall plot is more informative than the roc plot when evaluating binary classifiers on imbalanced datasets. *PLoS One* **10**, e0118432, DOI: 10.1371/journal.pone.0118432 (2015).
8. Simonyan, K., Vedaldi, A. & Zisserman, A. Deep inside convolutional networks: Visualising image classification models and saliency maps. *arXiv preprint arXiv:1312.6034* DOI: 10.48550/arXiv.1312.6034 (2013).
9. Zeiler, M. D. & Fergus, R. Visualizing and understanding convolutional networks. In *European Conference on Computer Vision*, 818–833, DOI: 10.1007/978-3-319-10590-1_53 (Springer, 2014).
